# Unveiling the Role of Protein Posttranslational Modifications in Glioma Prognosis

**DOI:** 10.1111/cns.70330

**Published:** 2025-03-16

**Authors:** Zhipeng Jiang, Hanxue Huang, Youwei Guo, Zihan Wang, Hailong Huang, Wen Yin, Haoxuan Huang, Lei Wang, Weidong Liu, Xingjun Jiang, Caiping Ren

**Affiliations:** ^1^ Department of Neurosurgery, Xiangya Hospital Central South University Changsha Hunan P.R. China; ^2^ Cancer Research Institute, Xiangya School of Basic Medical Science Central South University Changsha Hunan P.R. China; ^3^ National Clinical Research Center for Geriatric Disorders, Xiangya Hospital Central South University Changsha Hunan P.R. China; ^4^ Department of Clinical Pharmacology, Hunan Key Laboratory of Pharmacogenetics, and National Clinical Research Center for Geriatric Disorders, Xiangya Hospital Central South University Changsha P.R. China; ^5^ Institute of Clinical Pharmacology, Engineering Research Center for Applied Technology of Pharmacogenomics of Ministry of Education Central South University Changsha P.R. China; ^6^ The NHC Key Laboratory of Carcinogenesis and the Key Laboratory of Carcinogenesis and Cancer Invasion of the Chinese Ministry of Education Central South University Changsha Hunan P.R. China

**Keywords:** glioma, multiomics analysis, posttranslational modification, prognostic signature, *TOM1L1*

## Abstract

**Background:**

Gliomas represent the most aggressive malignancies of the central nervous system, with posttranslational modifications (PTMs) emerging as critical regulators of oncogenic processes through dynamic protein functional modulation. Despite their established role in tumor biology, the systematic characterization of PTM‐mediated molecular mechanisms driving glioma progression remains unexplored. This study aims to uncover the molecular mechanisms of glioma, with a focus on the role of PTMs.

**Methods:**

We analyzed the PTM pathway to classify glioma patients into distinct clusters. Comprehensive analyses compared intercluster differences in clinical outcomes, mutational landscapes, and immune microenvironment profiles. Differentially expressed genes (DEGs) were identified to construct a robust prognostic prediction model with machine learning approaches. Among the genes included in the model, *TOM1L1* (Target of Myb1 Like 1 Membrane Trafficking Protein) was selected for in vitro experimental validation to assess its role in glioma progression.

**Results:**

PTMs were found to influence glioma prognosis significantly. Dysregulation in specific pathways, such as glutathionylation and citrullination, was correlated with more aggressive clinical features. The prognostic model, comprising DEGs such as *TOM1L1*, demonstrated high predictive accuracy (c‐index = 0.867)—the scores derived from the model strongly correlated with glioma progression indicators. In vitro experiments revealed that TOM1L1 facilitates malignant progression by modulating PTM pathways, confirming its functional role in glioma.

**Conclusion:**

Our study establishes the first comprehensive PTM atlas in gliomas, revealing subtype‐specific modification patterns with clinical and therapeutic implications. *TOM1L1* emerges as a promising prognostic biomarker and a potential therapeutic intervention target. Targeting PTM pathways may offer novel strategies for glioma treatment, enhancing patient outcomes.

## Graphical abstract

Our work explores the role of protein posttranslational modifications (PTMs) in glioma. Through the screening of PTM pathways and the analysis of related molecular characteristics, we identified prognosis‐related genes and evaluated and constructed PTM‐related prognostic signatures.

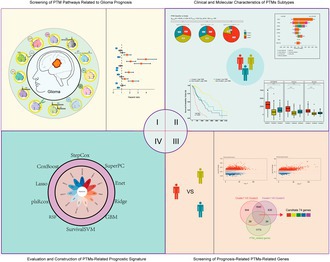

**Abbreviations**
CNV: copy number variation
DEGs: differentially expressed genes
DSS: disease‐specific survival rates
ESTIMATE: Estimation of STromal and Immune cells in MAlignant Tumors using Expression data
GSVA: gene set variation analysis
ME: mesenchymal
OS: overall survival
PN: proneural
PTMs: protein posttranslational modifications
ROC: receiver operating characteristic
RSF: random survival forest
superPC: supervised principal component analysis
TIDE: tumor immune dysfunction and exclusion
TMB: tumor mutation burden

## 1 Background

Glioblastoma (GBM) remains one of the most challenging diseases in neurosurgery, with previous molecular pathological treatment protocols proving largely ineffective [1]. To enhance perioperative treatment strategies in clinical practice, the WHO updated the classification of glioblastoma in 2021 by reintegrating histological and molecular features [2]. Critical indicators of poor prognosis in gliomas include *IDH* (Isocitrate Dehydrogenase) (NADP[+]) wild‐type status, absence of 1p/19q codeletion, and an unmethylated MGMT promoter [3]. However, these markers fail to fully capture the heterogeneity of glioma biology or guide targeted therapies effectively. Recent studies have explored novel approaches, including monoclonal antibodies and epigenetic modulators, yet clinical translation remains elusive [4]. This underscores the critical need to investigate understudied molecular mechanisms.

Posttranslational modifications (PTMs), which dynamically regulate protein function and tumor progression, including phosphorylation, ubiquitination, and acetylation, are pivotal in cancer pathogenesis by modulating protein stability, interactions, and signaling cascades [5, 6]. Aberrant PTMs have been implicated in tumor aggressiveness and therapeutic resistance. Acetylation, controlled by histone acetyltransferases (HATs) and histone deacetylases (HDACs), affects gene expression by modifying histones, thereby influencing chromatin structure and transcriptional activity. Dysregulated acetylation of histone and nonhistone proteins can alter critical cellular pathways, promoting cell growth, survival, and malignant transformation in cancer [7, 8]. Notably, hypoxia‐induced PAK1 acetylation at K420 inhibits dimerization while enhancing kinase activity, which subsequently phosphorylates ATG5 at T101 to drive GBM tumorigenesis via autophagy dysregulation [9]. Additionally, K‐acetylation represents a prominently dysregulated epigenetic modification in GBM compared with lower‐grade gliomas (LGG). Transcriptomic profiling identifies H3K14ac as the predominant acetylation signature (vs. H3K9ac), with distinct patterns correlating independently with patient survival, positioning acetylation as a prognostic biomarker candidate [10]. Ubiquitination represents an important PTM mechanism that governs protein fate and function through the ubiquitin–proteasome pathway. This process is orchestrated through a sequential three‐step enzymatic cascade involving the E1 ubiquitin‐activating enzyme, E2 ubiquitin‐conjugating enzyme, and E3 ubiquitin ligase. Beyond its canonical role in targeting proteins for proteasomal degradation, ubiquitination plays a versatile role in modulating protein subcellular localization, stability, and interaction networks, thereby influencing diverse cellular processes [11]. In GBM, the E3 ligase MAEA amplifies stemness and temozolomide resistance by mediating K48‐linked ubiquitination of PHD3 at K159, thereby stabilizing HIF‐1α under hypoxia [12]. Similarly, NEDD4‐driven polyubiquitination degrades the tumor suppressor TUSC2, fostering GBM progression through Bcl‐xL‐mediated antiapoptotic signaling [13]. Glycosylation is a critical PTM that involves the enzymatic attachment of sugar moieties to proteins or lipids. This alteration is crucial for protein folding, stability, and intercellular communication [14]. Glycosylation can modify specific proteins, affecting tumor cell stemness or altering the tumor microenvironment, ultimately promoting glioma progression [15, 16]. S‐nitrosylation is a redox‐associated posttranslational modification where nitroso (−NO) groups are added to protein cysteine residues. This modification is crucial for cellular processes such as signal transduction, protein stability, and enzyme activity [17].

In glioma, dysregulated PTMs drive therapeutic resistance and immune evasion through multifaceted mechanisms. For example, S‐nitrosylation can regulate the activation of microglia in the immune microenvironment, thereby influencing tumor progression [18]. Furthermore, the interplay between ERK1/2 S‐nitrosylation and ERK1/2 phosphorylation is involved in tumor cell development and the resistance of glioma cells to apoptosis [19]. Smith et al. further highlight the prognostic relevance of PTMs in glioma, demonstrating that stearoyl‐CoA desaturase (SCD) regulates DNA damage repair by modulating PARP1 activity—a process dependent on PARP1's PTM [20]. These works exemplify how PTMs can serve as both prognostic biomarkers and therapeutic targets across diverse molecular pathways. While previous studies have reported important roles of PTM‐related pathways in glioma, such as specific protein phosphorylation, ubiquitination, and other modifications, the systematic investigation of PTM s and their associated genes for glioma prognosis is relatively scarce. Given the complex role of PTMs in tumor biology, a deeper understanding of their impact on glioma progression may provide new therapeutic opportunities and prognostic biomarkers.

Our study initially focused on the heterogeneity of PTMs in gliomas to perform prognostic clustering and describe the immune and gene mutations associated with each cluster. Through clustering analysis, we identified distinct PTM profiles and subsequently developed a prognostic model incorporating PTM‐related genes, including *TOM1L1*, which demonstrated strong predictive power for patient survival outcomes. While TOM1L1 (Target of Myb1‐Like 1) has been implicated in endosomal trafficking and immune regulation [21, 22], its role in PTM‐mediated glioma progression remains entirely unexplored. This study exemplifies how PTMs can function as both prognostic biomarkers and therapeutic targets across diverse molecular pathways. By providing a systems‐level view of PTM interactions in gliomas, our research facilitates a more comprehensive understanding of these modifications. Furthermore, it offers new insights into the molecular processes driving glioma progression and identifies potential biomarkers that could guide the development of targeted treatment approaches.

## 2 Methods

### 2.1 Data Download and Preprocessing

Pathway‐related genes were initially identified through GSEA (https://www.gsea‐msigdb.org/gsea/index.jsp) by examining PTM pathways documented in the literature. A total of 19 pathways were integrated into our final analysis (Table S1). After consolidating the data, we calculated the associations between these pathways and glioma prognosis.

**FIGURE 1:**
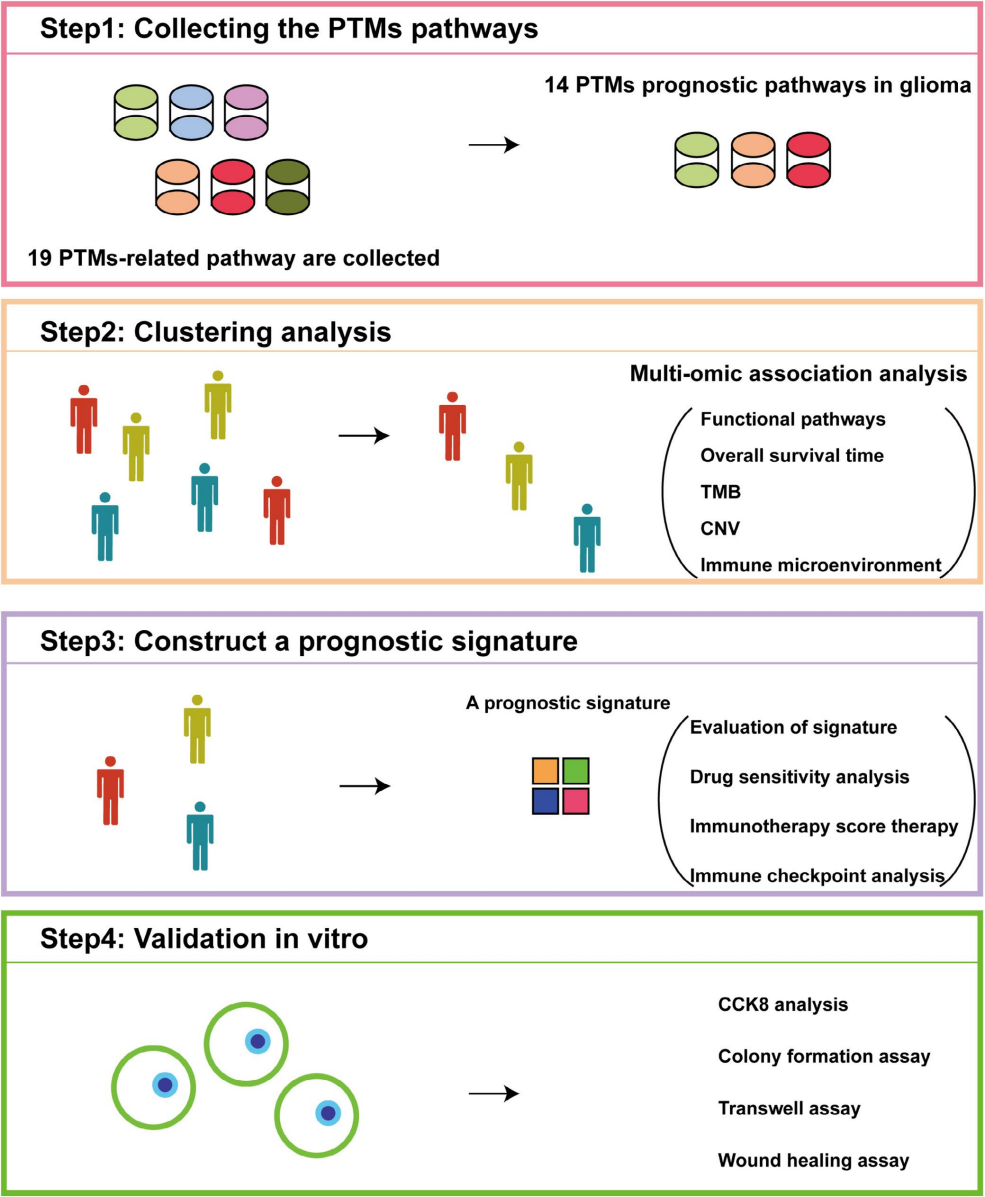
Experimental design and analytical workflow.

Glioma patient transcriptome and clinical data were obtained from The Cancer Genome Atlas (TCGA), the Chinese Glioma Genome Atlas (CGGA), and the Glioma Longitudinal AnalySiS (GLASS) Consortium. RNA‐Seq data were first converted into transcripts per million (TPM) to normalize for sequencing depth and gene length. Microarray data underwent SCAN normalization to correct for technical variability and batch effects. Following normalization, all datasets were log_2_‐transformed to stabilize variance and improve the suitability for downstream statistical analyses. An overview of the study design and analytical workflow is illustrated in Figure 1.

### 2.2 Unsupervised Clustering Analysis of Glioma Patients

Pathway scores corresponding to different PTM patterns in glioma patients were calculated using the “GSVA” R package. Subsequently, the “ConsensusClusterPlus” package was utilized to perform consensus clustering on these pathway scores, setting the number of repetitions to 500, using Pearson distance, and the Partitioning Around Medoids (PAM) algorithm. To determine the optimal number of clusters (*K*), we analyzed the cumulative distribution function (CDF) curves for *K* values ranging from 2 to 10. The optimal *K* was identified based on the “proportion of ambiguous clustering” (PAC) measure, we selected the *K* value where the CDF curve exhibited the smallest slope decrease within the PAC interval of 0.1–0.9, ensuring a robust and reliable clustering solution.

### 2.3 Survival Analysis and Functional Enrichment Analysis

We conducted survival analysis using the “survival” and “survminer” packages in R. Cox proportional hazards regression was performed to identify significant prognostic factors, and Kaplan–Meier survival curves were generated using “ggsurvplot” to visualize survival differences between patient groups. Receiver operating characteristic (ROC) analysis was conducted with the “timeROC” package to evaluate the predictive performance of the survival models. A nomogram integrating key clinical features such as age, tumor grade, and molecular markers was constructed using the “rms” package to facilitate individualized prognosis prediction.

Differential enrichment analysis between the cluster with the worst prognosis and the other clusters was performed using the GSVA method, followed by an integrated comparison of the differences.

### 2.4 Association Between PTM Clusters and Clinical Annotations

To explore the association between PTM classifier clusters and various clinical annotations, we employed the “ggstatsplot” package to generate pie chart visualizations. These charts depict the distribution of clinical features across the identified PTM clusters. Clinical annotations examined included grade, IDH status, 1p/19q codeletion, transcriptome subtype, and DNA methylation. The statistical significance of the associations between PTM clusters and clinical annotations was assessed using the chi‐squared test for categorical variables.

### 2.5 Description of Immune Characteristics and Analysis of Genetic Mutations

Immune cell infiltration in tumor tissues from GBM and LGG patients in the TCGA dataset was analyzed using two advanced single‐cell deconvolution algorithms, CIBERSORTX [23], and BayesPrism [24]. The CIBERSORTx deconvolution matrix was acquired from the TimeDB repository (https://timedb.deepomics.org/download). BayesPrism utilized single‐cell reference profiles from the CELLxGENE Portal (Accession: Core GBmap; https://cellxgene.cziscience.com/collections). To assess whether immune cell infiltration varied across distinct patient subgroups, immune infiltration data were integrated with cluster information derived from the three PTM subtypes. Differences in immune cell infiltration levels across the three clusters were evaluated using a one‐way analysis of variance. The ESTIMATE (Estimation of Stromal and Immune cells in Malignant Tumors using Expression data) algorithm was applied to calculate the proportions of immune cells in glioma patients, along with the Estimation (ESTIMATE) score, Immune score, and Stromal score. The “maftools” R package was used to analyze mutation profiles in glioma patients. Additionally, copy number variations (CNVs) in glioma samples were assessed using the GISTIC2.0 method.

### 2.6 Selection of Candidate Genes and Construction of the PTM‐Related Prognostic Signature

Differentially expressed genes (DEGs) were identified with a volcano map. Genes were deemed significant if they satisfied both a *p* value less than 0.01 and an absolute log_2_ fold change greater than 2. The overlapping DEGs were subsequently used to construct a prognostic linear model. Initial calculations were performed on the TCGA dataset, followed by further evaluation in the test sets (CGGA325 and CGGA693). The machine learning method was derived from Liu's literature [25]. To sum up, we employed a comprehensive integration of 10 machine learning algorithms: random survival forest (RSF), elastic network (Enet), Lasso, Ridge, stepwise Cox, CoxBoost, partial least squares regression for Cox (plsRcox), supervised principal components (SuperPC), generalized boosted regression modeling (GBM), and survival support vector machine (survival SVM). Feature selection‐capable algorithms (Lasso, stepwise Cox, CoxBoost, RSF) were prioritized to generate a consensus model. A total of 101 algorithm combinations were systematically evaluated under a leave‐one‐out crossvalidation (LOOCV) framework. The optimal algorithm was then applied to calculate the PTM score for each patient. Patients from multiple databases were scored based on this signature and then divided into high‐ and low‐score groups according to the median score for the following analysis.

### 2.7 Drug Sensitivity and Immune Evasion Analysis

Drug sensitivity for each medication was calculated using the R packages “pRRophetic” and “oncoPredict,” and differences in IC50 values between groups were visualized using bar plots. Immune evasion mechanisms, including “Dysfunction,” “IFNG,” “Exclusion,” “MSI,” and “TIDE,” were assessed between groups using the TIDE database (http://tide.dfci.harvard.edu/). Gene expression data related to immune pathways were extracted from the TCGA, CGGA, and GLASS datasets and integrated with clinical metadata. The immune‐related gene expression data were preprocessed and scaled. Samples were then grouped based on previously calculated risk scores (PTM score^high^ and PTM score^low^) to assess differences in immune characteristics between these groups. The integration of gene expression and clinical metadata was used to analyze immune features across the distinct risk score categories.

### 2.8 Experiments on Tumor Cells Exhibiting Malignant Phenotype

The glioma cell lines LN229 and U251 were treated with si‐RNA to evaluate its impact on RNA expression, followed by experiments to assess malignant phenotypes. The *TOM1L1* sequence design includes a forward primer of 5′‐GCTTCCCAGGAGGTGTGGAT‐3′ and a reverse primer of 5′‐TGAGGGAGGAAACTGAACGC‐3′.

Next, we assessed the changes in cell proliferation following transfection using the CCK‐8 assay and colony formation assay. Suspended tumor cells were evenly distributed at 2000–4000 cells per well in a 96‐well plate. Cell numbers were measured every 24 h, and after adding diluted CCK‐8 solution, the absorbance was recorded after 1 h. For the colony formation assay, 1000–2000 cells were seeded per well in a six‐well plate, ensuring the cells were dispersed into single cells to facilitate the formation of single clones. After 1–2 weeks, the cells were fixed and stained with crystal violet, and colonies were counted to evaluate the changes in proliferation.

The Transwell assay was used to evaluate cell invasion ability. Matrigel was precoated in the upper chamber of the Transwell insert, and 20,000 cells were resuspended in serum‐free medium and plated. The lower chamber contained a complete medium. After 24–72 h, cells were counted under a microscope. Cell migration was evaluated after 48–72 h of incubation using crystal violet staining and photography to identify differences in invasion capability.

Tumor cells in a six‐well plate were grown to confluence for wound healing assays. A 200 μL pipette tip was used to create a scratch and remove detached cells. Cell migration distances were measured using microscope images captured at 0 and 48h intervals.

### 2.9 Statistical Methods and Data Analysis

All statistical analyses were done using R version 4.2.2 and the appropriate Bioconductor packages. Data analysis was conducted with GraphPad Prism 8 (GraphPad Software Inc.). Continuous variables were initially subjected to normality testing to determine appropriate statistical approaches. For normally distributed data, two‐group comparisons were conducted using Student's *t*‐test, while one‐way analysis of variance (ANOVA) was applied for three groups. In non‐normally distributed data, the Mann–Whitney *U*‐test and Kruskal–Wallis *H*‐test served as nonparametric alternatives for two‐group and three‐group comparisons, respectively. Categorical variables were analyzed via Fisher's exact test, and survival differences were assessed using the log‐rank test. All statistical tests were two‐sided, with a significance threshold of *p* < 0.05. To standardize RNA‐seq data from the TCGA cohort, raw read counts were converted to transcripts per kilobase million (TPM) and log_2_‐transformed with the formula log_2_ (TPM + 1), ensuring variance stabilization and compatibility with downstream molecular analyses.

## 3 Results

### 3.1 Identification of Prognostic PTM Pathways in Glioma

**FIGURE 2:**
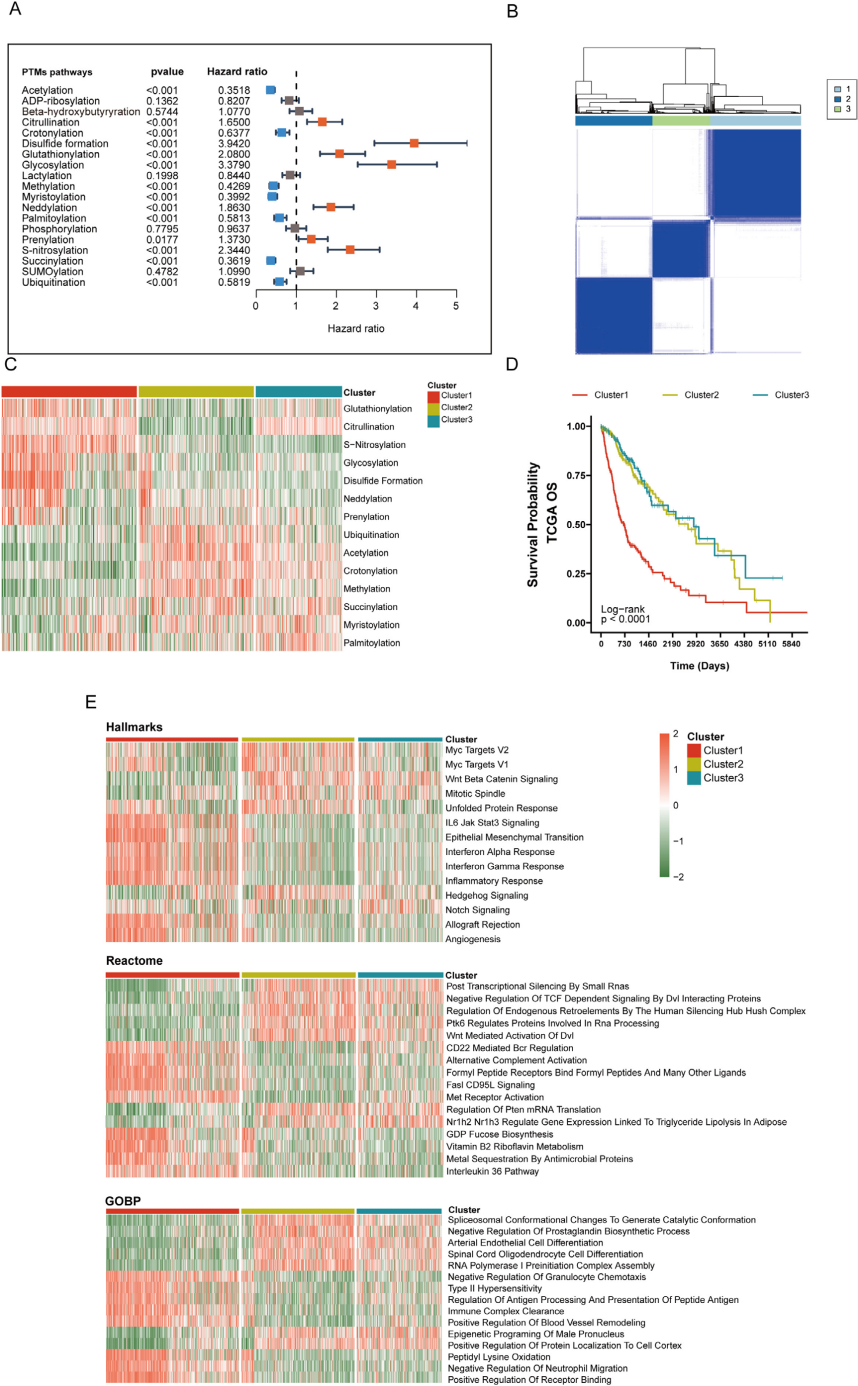
Cluster glioma patients based on PTM pathway expression profiles. (A) Identification of PTM pathways associated with glioma prognosis. (B) Divide glioma patients into the most suitable clusters (*k* = 3). (C) Heatmap visualization of PTM‐related pathways. (D) Cluster 1 exhibits the poorest prognostic clinical characteristics. (E) Results of the HALLMARK, Reactome, and GOBP enrichment analysis.

We performed GSVA analysis to evaluate PTM pathway activities across TCGA glioma samples. Among all the assessed pathways, 14 PTM pathways were significantly associated with patient prognosis (Figure 2A). An unsupervised clustering analysis of these prognostic pathways revealed three distinct molecular subtypes (optimal cluster number *k* = 3, Figure 2B and Figure S1A–F). The three subtypes demonstrated unique PTM pathway signatures: Cluster 1 was characterized by elevated activities in glutathionylation, citrullination, and S‐nitrosylation pathways, while Clusters 2 and 3 showed distinct patterns in ubiquitination, acetylation, and crotonylation pathways (Figure 2C).

### 3.2 Prognostic and Biological Characterization of PTM Clusters

Survival analysis revealed significant prognostic differences among the subtypes, with Cluster 1 showing significantly worse overall survival (OS) and disease‐specific survival (DSS) compared to other clusters (Figure 2D and Figure S1G). We performed a comprehensive pathway enrichment analysis using multiple databases to explore the biological implications of these PTM‐based subtypes. In the Hallmarks database, Cluster 1 showed significant enrichment in inflammatory pathways including Interferon *α*/*γ* Response and IL6/JAK/STAT3 signaling, as well as epithelial–mesenchymal transition. Consistently, analysis using the Reactome database revealed enrichment of immune‐related pathways in Cluster 1, such as CD22‐mediated BCR regulation and alternative complement activation. Gene Ontology Biological Process (GOBP) analysis further supported the immune‐regulatory characteristics of Cluster 1, showing enrichment in pathways related to immune complex clearance and regulation of antigen processing and presentation (Figure 2E).

### 3.3 Distinct Clinical and Immunological Profiles of PTM Clusters

**FIGURE 3:**
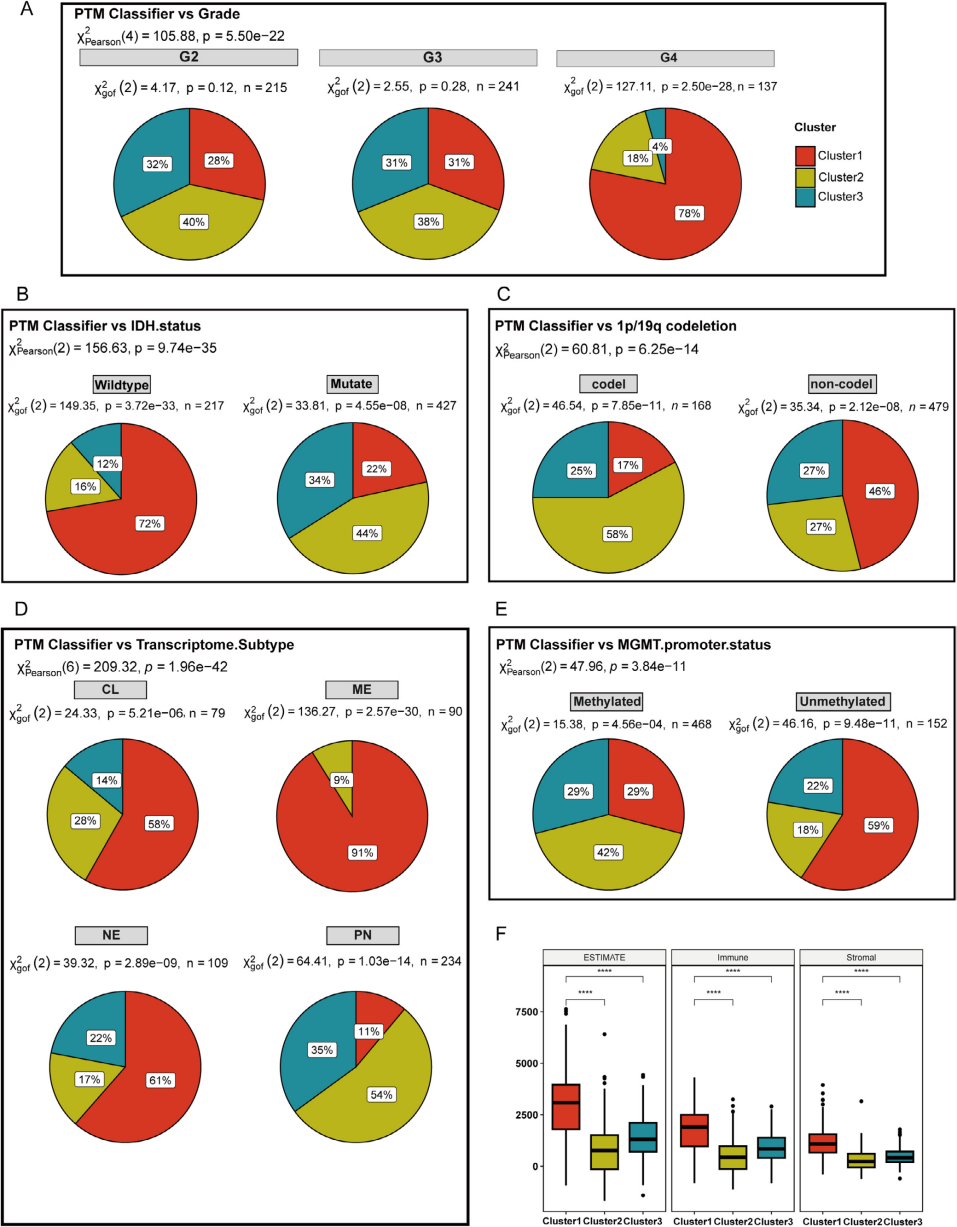
Clinical and molecular profiling of glioma patients based on PTM Classifiers. (A) Distribution of glioma grades across different PTM clusters. (B) Distribution of IDH mutation status across PTM clusters. (C) Distribution of 1p/19q codeletion across PTM clusters (codel, codeletion; noncodel, no codeletion). (D) Distribution of transcriptome subtype across PTM clusters (CL, classical; ME, mesenchymal; NE, neural; PN, proneural). (E) Distribution of MGMT promoter methylation status across PTM clusters. (F) ESTIMATE scores across PTM clusters. ****p < 0.0001.

We investigated the correlation between each cluster and various glioma subtypes associated with prognosis, including tumor grade, IDH status, 1p/19q codeletion status, transcriptome subtype, and MGMT promoter methylation status. As illustrated in Figure 3A, Cluster 1, associated with the poorest prognosis, was predominantly found in Grade 4 gliomas (proportion = 78%, *p* = 2.5 × 10^−28^). In contrast, the proportions of Grade 2 and Grade 3 gliomas did not show significant differences (*p* = 0.12 and *p* = 0.28, respectively). Notably, among IDH wild‐type gliomas, Cluster 1 constituted the largest proportion, accounting for 72%, whereas it comprised only 22% of IDH mutant gliomas (Figure 3B). In terms of 1p/19q codeletion status (Figure 3C), Cluster 1 was most prevalent in noncodeleted gliomas (46%) and was least pervasive in codeleted gliomas (17%) (*p* = 2.12 × 10^−8^ and *p* = 7.85 × 10^−11^, respectively). Regarding transcriptome subtypes, Cluster 1 was predominantly associated with the mesenchymal (ME) subtype (proportion = 91%, *p* = 2.57 × 10^−30^). Conversely, Clusters 2 and 3 were more frequently observed in the proneural (PN) subtype, with proportions of 54% and 35%, respectively (*p* = 1.03 × 10^−14^) (Figure 3D). MGMT promoter methylation is an independent prognostic predictor for glioma patients. The median survival time of patients with methylation positivity is usually longer than that of patients with methylation negativity. In our results, Cluster 1 exhibited a higher proportion in unmethylated status (proportion = 59%, *p* = 9.48 × 10^−11^), while Cluster 2 comprised the fewest proportion of unmethylated status (18%) and highest proportions in methylated status (42%) (Figure 3E). Cluster 1 reflects a convergence of several high‐risk factors, strongly associated with aggressive disease biology. It is predominantly found in Grade 4 gliomas, particularly those that are IDH‐wildtype, 1p/19q noncodeleted, mesenchymal subtype, and MGMT unmethylated. In contrast to Clusters 2 and 3, which are enriched with more favorable markers (such as IDH mutations in 78% vs. 22%, 1p/19q codeletion in 83% vs. 17%, and MGMT methylation in 29% vs. 71%), Cluster 1 patients lack these protective features, correlating with significantly shorter survival. Additionally, the immunosuppressive, stroma‐rich microenvironment in Cluster 1, marked by dense extracellular matrix deposition, may present both physical and biochemical barriers to conventional therapies.

We examined the immune and stromal components to investigate the tumor microenvironment across various clusters further. As shown in Figure 3F, Cluster 1 demonstrates a significantly higher ESTIMATE score than Clusters 2 and 3. This finding suggests that tumors in Cluster 1 exhibit more extensive interactions with the immune system and the extracellular matrix. The Immune score analysis aligns with the ESTIMATE score, showing elevated expression levels of immune‐related genes in Cluster 1. Additionally, the Stromal score indicates that Cluster 1 has higher stromal cell infiltration within the tumor microenvironment. This observation highlights that the extracellular matrix in Cluster 1 tumors is particularly dense, which may contribute to tumor growth and metastasis by providing structural support. Furthermore, the dense extracellular matrix might serve as a physical barrier to immune cell infiltration, potentially reducing the effectiveness of antitumor therapies.

### 3.4 Molecular Mutation Landscape and Immune Microenvironment in Glioma Clusters

**FIGURE 4:**
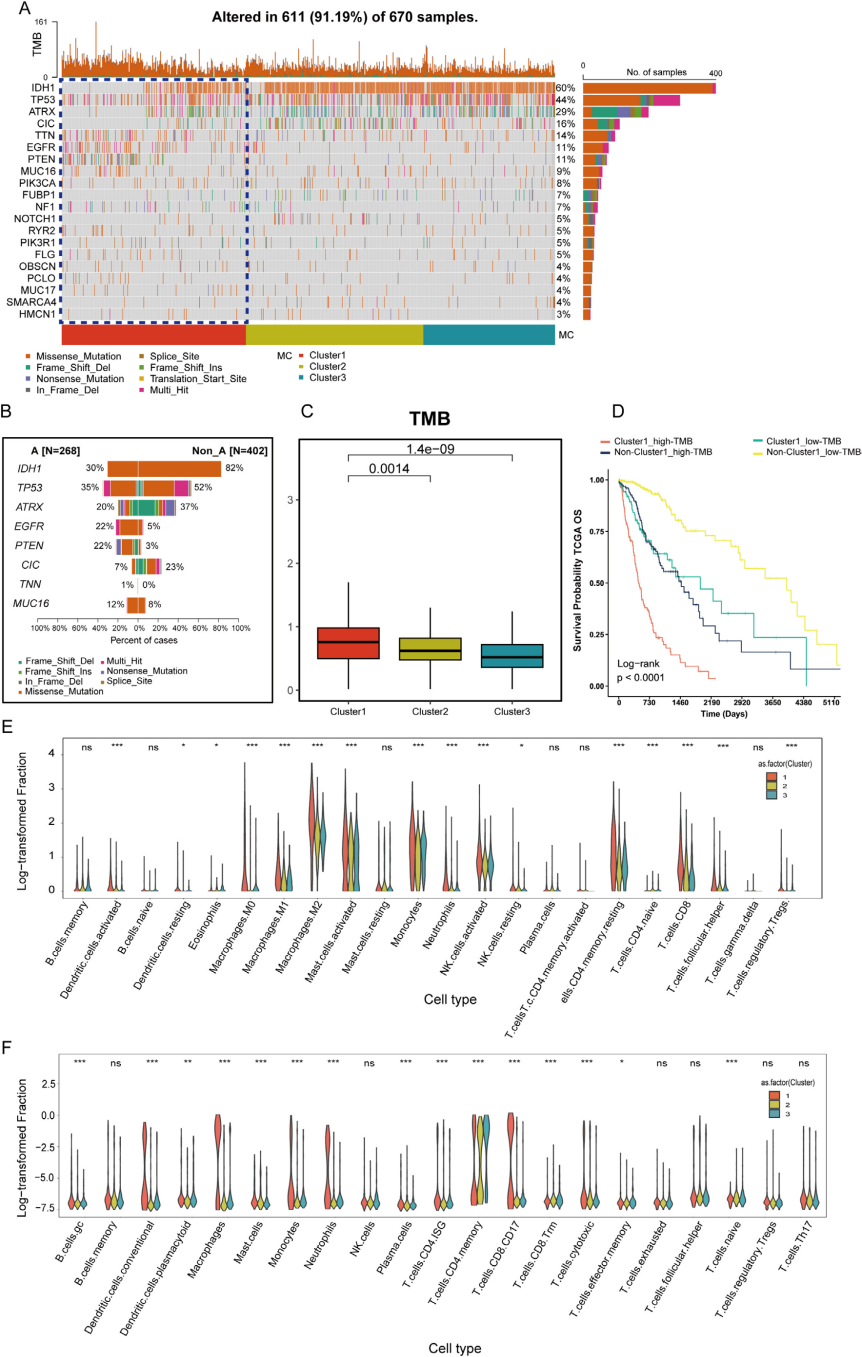
Genomic alterations and TMB across PTM clusters. (A) Overview of genetic alterations in glioma samples. (B) Comparison of mutation frequencies between two distinct groups. (C) Distribution of tumor mutational burden (TMB) across PTM clusters. (D) Kaplan–Meier survival analysis stratified by TMB and cluster grouping. (E) Immune cell composition across clusters analyzed using CIBERSORTx. (F) Immune cell composition across clusters analyzed using BayesPrism. ns, no statistical significance; *p < 0.05; **p < 0.01; ***p < 0.001.

Tumor mutational burden (TMB) was calculated as the total number of somatic mutations (including single nucleotide variants and indels) per megabase (Mb) of the genome. TMB is a well‐established biomarker that reflects the overall level of genomic instability and is associated with response to immune checkpoint inhibitors in various cancers, including glioma [26, 27]. Firstly, we analyzed the TMB across 670 glioma samples and detected mutations in 611 cases (91.19%). Specifically, *IDH1* (Isocitrate Dehydrogenase 1), *TP53* (Tumor Protein P53), and *ATRX* (ATRX Chromatin Remodeler) were the most frequently mutated genes among the glioma samples. Interestingly, patients in Cluster 1 carried fewer mutations in *IDH1*, *TP53*, *ATRX*, and *CIC* (Capicua transcriptional repressor) but exhibited higher overall mutations in *EGFR* (epidermal growth factor receptor) and *PTEN* (phosphatase and tensin homolog), reflecting their poor prognosis (Figure 4A,B). We also analyzed the TMB and found that Cluster 1 had a significantly higher TMB compared to Clusters 2 and 3 (*p* = 0.0014 and *p* = 1.4 × 10^−9^, respectively) (Figure 4C). In Cluster 1, the high‐TMB group exhibited the poorest prognosis, whereas the low‐TMB group outside Cluster 1 showed the most favorable prognosis (Figure 4D).

Next, we used violin plots to illustrate the proportions of various immune cells calculated by two single‐cell deconvolution algorithms—CIBERSORTx and BayesPrism. The violin plots display log_10_‐transformed fractions of various immune cell subtypes, with statistical significance of group‐wise comparisons indicated above each cell type. This includes markedly elevated infiltration of Macrophages M0, M1, and M2, which are well‐known contributors to tumor progression and immunosuppression within the tumor microenvironment. Additionally, higher levels of activated immune cells, such as CD8^+^ T cells and T follicular helper cells, suggest active immune engagement. Conversely, lower levels of resting immune cells, such as Dendritic cells resting and NK cells resting, indicate a shift in the immune system toward activation or exhaustion (Figure 4E). This pattern was consistently observed when employing BayesPrism (Figure 4F), where macrophage populations, as well as CD4 memory T cells, cytotoxic T cells, and exhausted T cells, were significantly enriched in Cluster 1 relative to the other clusters. These findings underscore the distinct immune microenvironment associated with Cluster 1, suggesting that these immune cell populations may play a pivotal role in driving the tumor's biological and clinical behavior.

### 3.5 Machine Learning‐Driven Construction of PTM‐Related Prognostic Models

**FIGURE 5:**
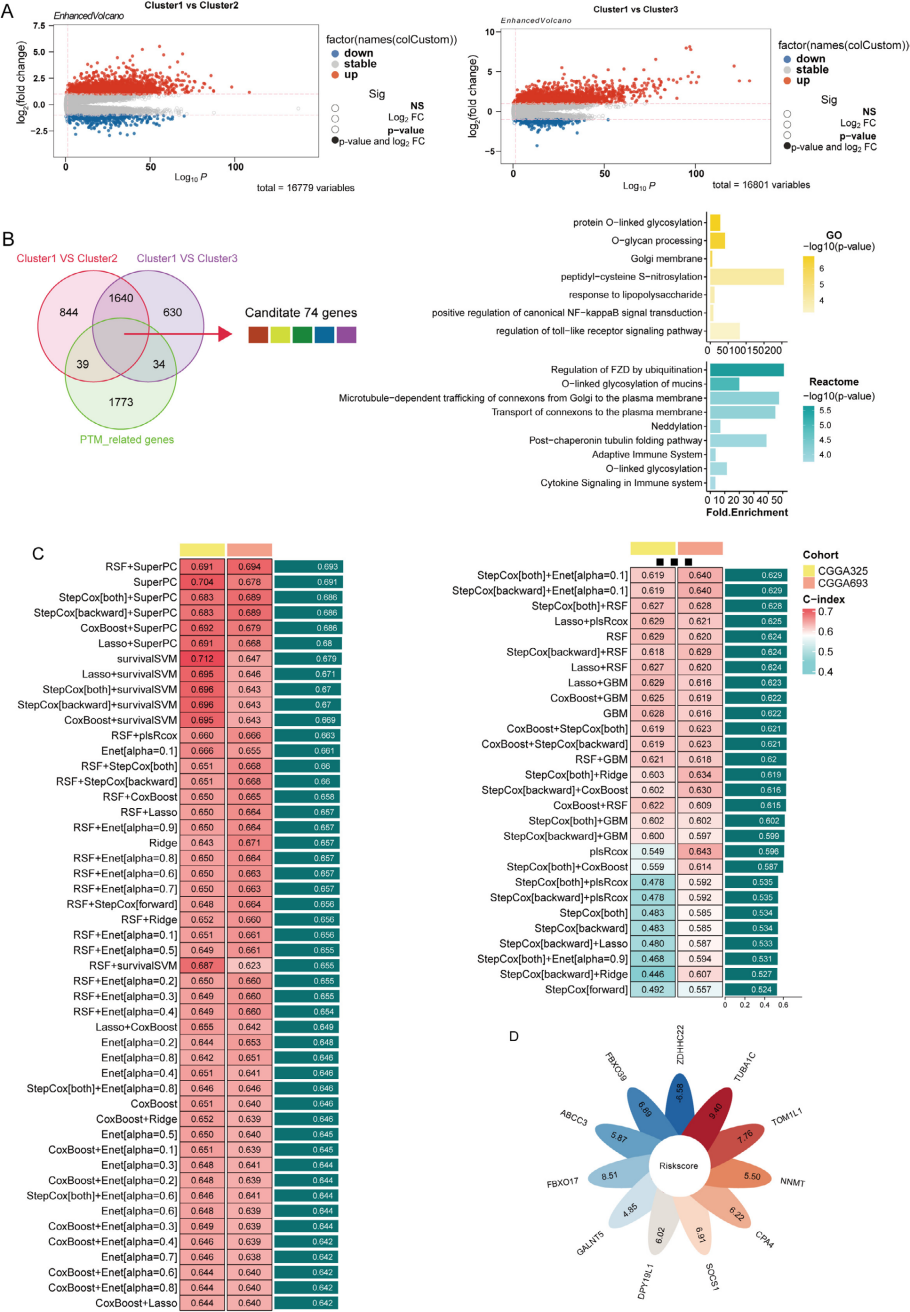
Selection of PTM prognostic genes and construction of PTM‐associated prognostic signature. (A) Volcano plots illustrating differential gene expression between Cluster 1 and Cluster 2 (left) and Cluster 1 and Cluster 3 (right). (B) Venn diagram showing the overlap of differentially expressed PTM‐related prognostic genes across comparisons (left) and GO/Reactome enrichment analysis based on DEGs. (C) Heatmap summarizing the performance of various feature selection models across different cohorts (CGGA325, CGGA693) using C‐index scores. (D) Feature scores for the top selected 11 genes identified through the RSF + SuperPC model.

Next, we identified the differentially expressed genes among the three clusters and visualized the results using a volcano plot (Figure 5A). Subsequently, PTM‐related candidate genes were filtered for the prognostic signature. As illustrated in Figure 5B, Cluster 1 exhibited 2597 DEGs compared to Cluster 2 and 2378 genes compared to Cluster 3. Incorporating PTM‐related genes into the differential expression analysis, we identified 74 genes across these comparisons. Differential gene enrichment analysis identified significant enrichment in two PTM‐related pathways: Protein O‐linked glycosylation and O‐glycan processing and peptidyl‐cysteine S‐nitrosylation. Additionally, pathways involved in the regulation of Toll‐like receptor signaling and the positive regulation of canonical NF‐κB signal transduction were also significantly enriched. These findings suggest that the differentially expressed genes play crucial roles in immune responses and tumor‐immune interactions. Consistent with these results, enrichment analysis of Reactome pathways revealed similar patterns, with significantly enriched pathways including cytokine signaling in the immune system, O‐linked glycosylation, and O‐linked glycosylation of mucins.

We further applied multicombinational machine learning analysis to these 74 differentially expressed genes. Among the various algorithms tested, the combination of random survival forest (RSF) and supervised principal component analysis (superPC) demonstrated superior performance (Figure 5C). This approach enabled us to identify 11 key genes (Table S2), which we subsequently used to construct a PTM‐related prognostic signature (Figure 5D).

### 3.6 Comprehensive Evaluation of PTM‐Related Signature Validity

**FIGURE 6:**
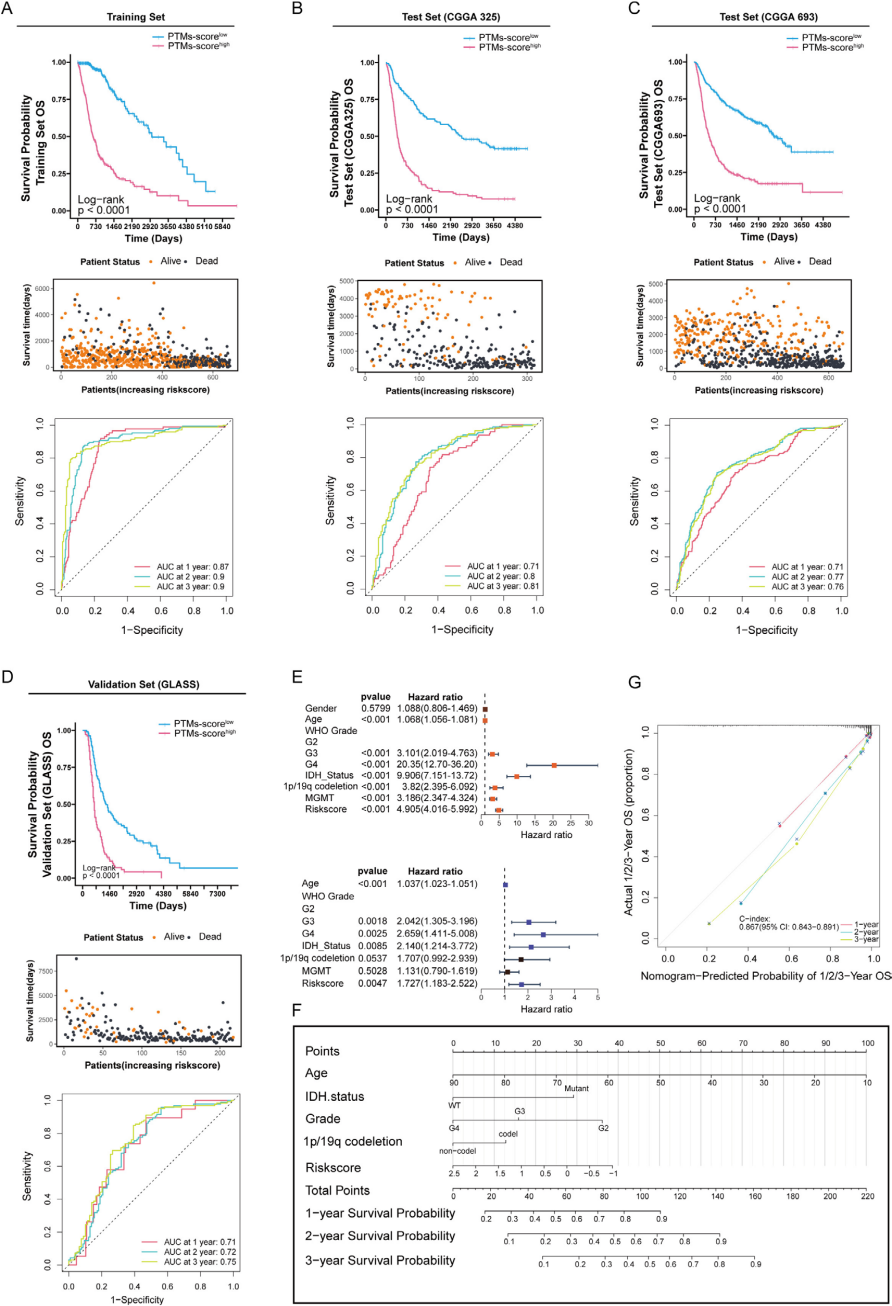
Validation of PTM‐related prognostic signature in multiple cohorts. (A–D) Kaplan–Meier survival curves for the training set, comparing OS between high and low PTM‐score groups. The scatter plot shows patient status (alive vs. dead) according to increasing risk scores, and the ROC curve depicts model sensitivity and specificity at 1, 2, and 3 years, with area under the curve (AUC) values. (E) Univariate and multivariate Cox regression analyses of clinical features and PTM‐based risk score in predicting OS. (F) Nomogram integrating PTM risk score with clinical factors (e.g., grade, IDH status) to estimate 1‐, 2‐, and 3‐year survival probabilities. (G) Calibration curve assessing the accuracy of the nomogram's predicted survival probabilities at 1, 2, and 3 years.

We further comprehensively evaluated the validity and accuracy of the PTM‐related signature using multiple metrics. A patient‐specific risk score was calculated and stratified into low‐ and high‐risk groups using the median value as the discriminatory threshold. In the TCGA training set, patients with high PTM scores demonstrated significantly lower average survival rates than those with low scores, as shown in the accompanying scatter plot of patient statuses. The ROC curves demonstrated AUC values of 0.87, 0.90, and 0.90 for 1‐, 2‐, and 3‐year survival predictions, respectively, indicating the model's exceptional proficiency in forecasting survival outcomes (Figure 6A). The validation sets (CGGA325 and CGGA693) confirmed the high‐PTM score group's shorter OS, supported by AUC values indicating strong prognostic assessment (Figure 6B,C). We broadened the validation scope by including the GLASS datasets as supplementary verification sets. Kaplan–Meier curves (Figure 6D) demonstrate that patients with high PTM scores consistently exhibit significantly shorter survival times compared to those with low PTM scores, indicating clear prognostic differences. After performing univariate and multivariate Cox analysis, we identified five independent prognostic factors, including age, glioma grade, and risk score (Figure 6E). These factors were then used to construct a nomogram to predict the prognosis of glioma cases. High age, IDH wild status, high grade, and adverse clinical–pathological features were associated with reduced 1‐, 2‐, and 3‐year survival probabilities (Figure 6F). Our analysis yielded a concordance index (c‐index) of 0.867 (0.843–0.891), reflecting robust prognostic prediction accuracy (Figure 6G).

### 3.7 Exploring the Connection Between PTMs, Drug Sensitivity, and Glioma Treatment Outcomes

**FIGURE 7:**
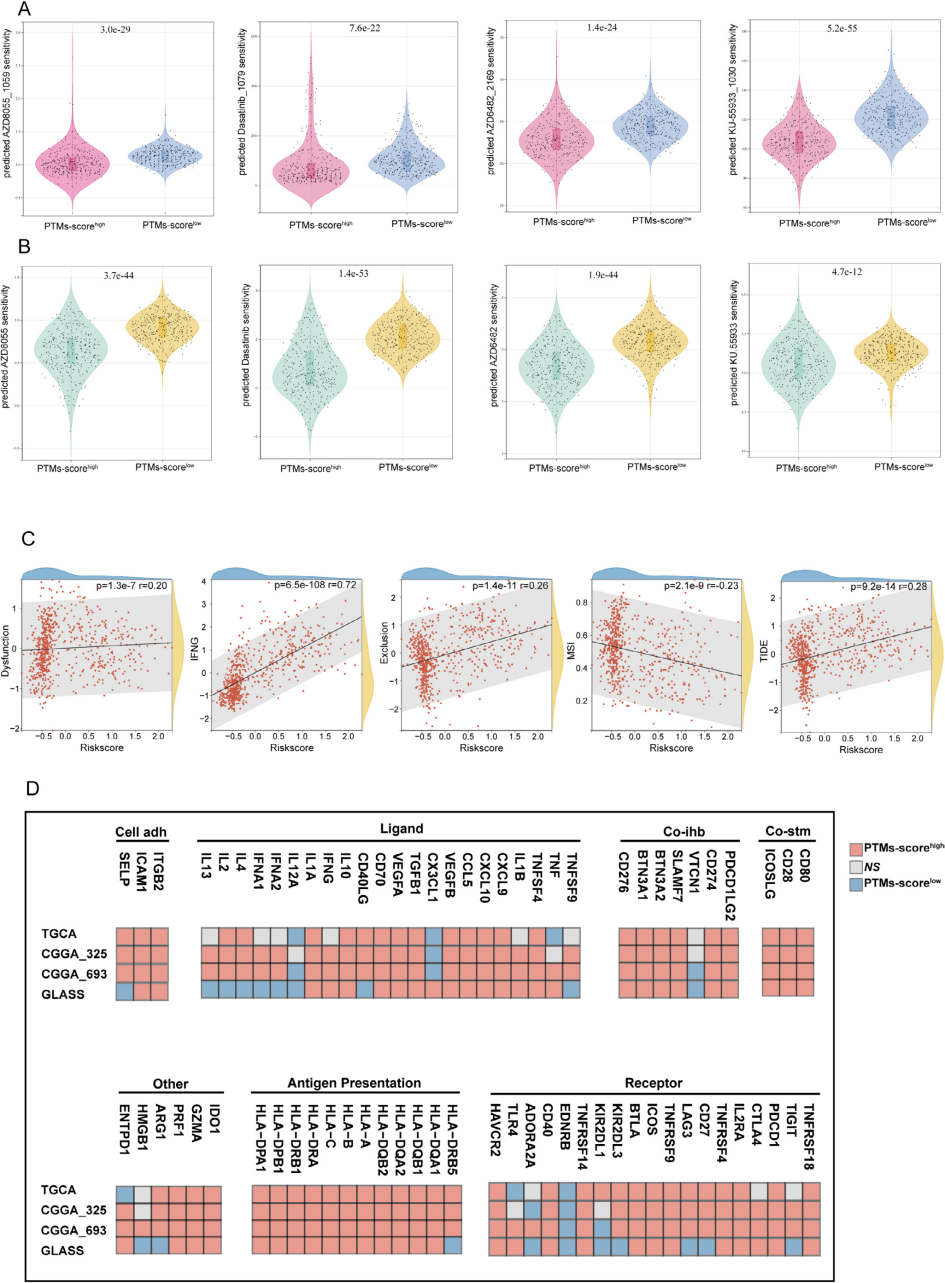
Correlation of PTM scores with drug sensitivity, TIDE scores, and immune checkpoint features. (A) Differential drug sensitivity profiles between high and low PTM‐score cohorts for clinically actionable compounds (oncopredict R packages). (B) Differential drug sensitivity profiles between high and low PTM‐score cohorts for clinically actionable compounds (pRRophetic R packages). (C) Scatter plot comparing immune‐related metrics, dysfunction scores, IFNG, exclusion, MSI (microsatellite instability), and TIDE scores (tumor immune dysfunction and exclusion). (D) Heatmap of immune‐related gene expressions across PTM‐score groups. High age, IDH wild status, high grade, and adverse clinical–pathological features were associated with reduced 1‐, 2‐, and 3‐year survival probabilities (cell adh, cell adhesion; Co‐ihb Co‐inhibitor; Co‐stm Co‐stimulator).

We investigated the clinical relevance of this biomarker in relation to treatment response. By analyzing drug sensitivity in TCGA glioma patients using the OncoPredict and pRRophetic packages, we identified four drugs that exhibited a strong association with the PTM score across both training datasets: AZD8055, Dasatinib, AZD6482, and KU‐55933. The results demonstrated that these drugs showed significantly higher predicted sensitivity in the high PTM‐score group, highlighting a potential relationship between PTM score and drug efficacy (Figure 7A,B).

To further explore the relationship between PTM score and immune therapy sensitivity‐related indicators, we conducted a correlation analysis with metrics such as dysfunction, interferon‐gamma (IFNG), exclusion, microsatellite instability (MSI), and the tumor immune dysfunction and exclusion (TIDE) score. As shown in Figure 7C, the PTM score was positively correlated with the TIDE score, dysfunction, exclusion, and IFNG levels. These findings suggest that a high PTM score is associated with immune resistance mechanisms, including impaired immune cell functionality, exclusion of immune cells from the tumor microenvironment, and increased inflammatory signaling. These characteristics collectively indicate a higher likelihood of immune evasion and resistance to immunotherapy. In contrast, the PTM score was negatively correlated with the MSI score, implying that tumors with a high PTM score may exhibit poor responsiveness to immunotherapy. This negative correlation aligns with the fact that lower MSI is typically associated with a reduced mutational burden, limiting the neoantigens available for immune recognition.

Additionally, to evaluate the expression patterns and functional associations of immune‐related molecules across different datasets, we stratified RNA‐seq data from four research cohorts (TCGA, CGGA325, CGGA693, and GLASS) based on PTM scores. In the high PTM‐score group, a substantial number of molecules across various immune modulator categories, including ligands, receptors, costimulatory molecules, and antigen‐presenting genes, were positively correlated with the PTM score. For instance, genes involved in antigen presentation, such as HLA‐A and HLA‐B, and co‐stimulatory molecules, such as CD80 and CD28, showed enhanced expression, indicating a potential activation of immune pathways in tumors with high PTM scores. However, certain molecules, such as VTCN1 (V‐Set domain‐containing T‐cell activation inhibitor 1), IL12A (interleukin‐12A), and EDNRB (endothelin receptor Type B), exhibited significant negative correlations with PTM score (Figure 7D). These findings suggest that while high PTM scores may enhance specific immune activation pathways, they might simultaneously downregulate others, further emphasizing the complex immune modulation in these tumors.

### 3.8 Role of PTM‐Related Gene TOM1L1 in Glioblastoma Progression

Based on the importance scores from our machine learning model, *TUBA1C*, *FBXO17*, and *TOM1L1* emerged as the top three genes. While *TUBA1C* and *FBXO17* have been extensively studied in glioma and are known to promote tumor progression, *TOM1L1*'s role in glioma remains underexplored, particularly in the context of PTM‐mediated mechanisms [28, 29].

**FIGURE 8:**
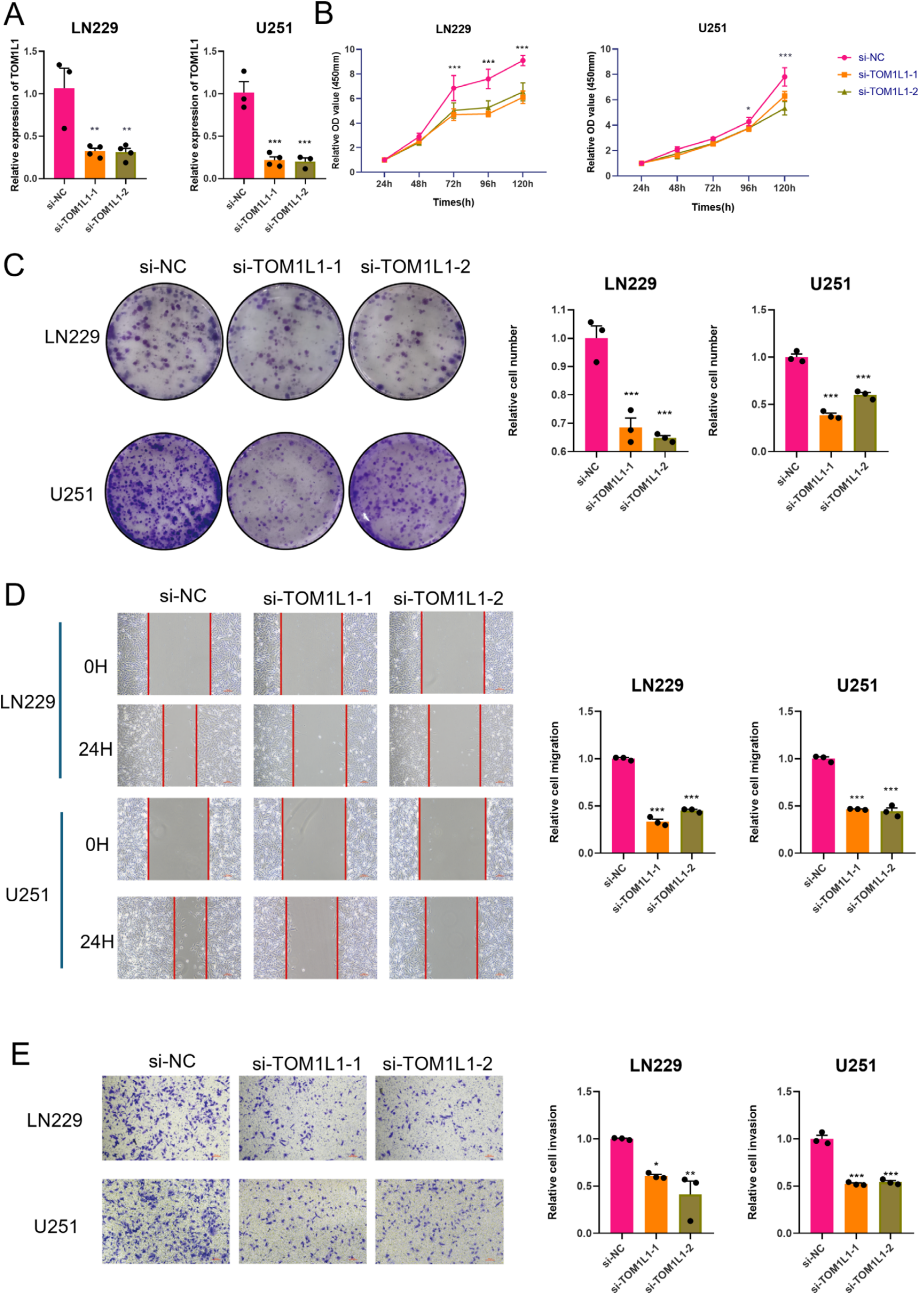
Impact of TOM1L1 knockdown on malignant phenotypes in glioblastoma cells. (A) qPCR analysis to evaluate the knockdown efficiency of si‐TOM1L1 in glioblastoma cells LN229 and U251. (B) The effect of TOM1L1 knockdown on glioblastoma cell proliferation was assessed using a CCK8 assay. (C) Colony formation assay in LN229 and U251 cells following TOM1L1 knockdown. (D) Scratch assay showing cell migration after TOM1L1 knockdown in glioblastoma cells. (E) Transwell assay illustrating cell invasion following TOM1L1 knockdown. *p < 0.05; **p < 0.01; ***p < 0.001.

To investigate the functional role of PTM‐related prognostic genes in glioblastoma, we conducted a series of experiments targeting *TOM1L1*, a gene believed to impact the malignancy of tumors potentially. The LN229 and U251 GBM cell lines were selected for this study based on two criteria. First, bioinformatic analysis of the Cancer Cell Line Encyclopedia (CCLE) revealed these cell lines among the top *TOM1L1*‐expressing glioblastoma models, ensuring physiological relevance for functional studies (Figure S1H). Second, both cell lines are extensively characterized and widely employed in GBM research, particularly for investigating tumor invasion, stemness, and resistance mechanisms. We evaluated the effects of *TOM1L1* knockdown by qPCR (Figure 8A). The CCK8 assay demonstrated that knockdown of *TOM1L1* significantly reduced the proliferation rate of malignant cells (Figure 8B). The colony formation assay showed that the number and size of colonies were markedly suppressed after *TOM1L1* knockdown (Figure 8C). These results demonstrate that TOM1L1 is essential for sustaining glioma cell proliferation and survival. Additionally, the scratch assay confirmed that *TOM1L1* promotes the migratory ability of glioma tumor cells (Figure 8D). Finally, the Transwell assay indicated that *TOM1L1* effectively enhances the invasion capacity of malignant cells (Figure 8E). These results generally indicate that the knockdown of *TOM1L1* significantly impaired the proliferation, colony formation, migration, and invasion of glioblastoma cells, suggesting that *TOM1L1* may act as an oncogenic driver of glioblastoma by promoting invasive tumor phenotypes.

## 4 Discussion

PTMs are mechanisms occurring after protein synthesis, implicated in the progression of diseases such as cancer and metabolic disorders. Glioma, a highly invasive malignant tumor, poses significant challenges in diagnosis and treatment due to its poor prognosis, attracting considerable clinical attention [30]. This study systematically analyzed the interaction between PTM mechanisms and glioma using distinct PTM‐associated pathways. Our findings suggest that different types of PTMs collectively influence glioma prognosis. We identified prognostically relevant genes, including *TOM1L1*, which may influence malignant cell progression by modulating various PTM pathways. Our study enhances the understanding of glioma pathophysiology and offers a new perspective with potential clinical significance for guiding treatment strategies and prognostic evaluation.

Our classification approach, based on PTM pathways, underscores the heterogeneity of gliomas. The identification of three distinct clusters with varying PTM pathways activity and prognoses highlights the complexity of glioma biology. Cluster 1, characterized by elevated expression of pathways such as glutathionylation, citrullination, and S‐nitrosylation, demonstrated the worst prognosis, evidenced by reduced overall and disease‐specific survival rates. The aberrant expression of these PTM pathways may lead to abnormal activation or inhibition of proteins, resulting in alterations in the cellular state within the microenvironment, which can, in turn, drive tumor initiation and progression. For example, glutathionylation, S‐nitrosylation, and disulfide formation are critical pathways involved in redox regulation [31]. In the context of glioma, the elevated activity of these pathways may enable tumor cells to withstand better oxidative stress, a common feature of the tumor microenvironment due to increased metabolic activity and immune responses. This adaptive advantage could facilitate the survival and proliferation of more aggressive tumor cells in Cluster 1, contributing to its poorer clinical outcomes.

Molecular mutation and tumor immunity are crucial prognostic indicators of cancer. Cluster 1 exhibits high immune‐related scores, including ESTIMATE, immune, and stromal scores, with a corresponding decrease in tumor purity. Algorithmic analysis of tumor microenvironment cell infiltration indicates it is associated with a highly immune‐active yet potentially dysfunctional tumor microenvironment. Elevated macrophage activity (M0, M1, M2) and exhausted T cells imply that immunosuppression and immune escape mechanisms play a central role in the aggressiveness of this subtype. These cell types have been linked to glioma progression [32]. Cluster 1 shows an elevated TMB score, reflecting a higher frequency of genetic mutations in the tumor. Our findings align with previous research indicating that high TMB is frequently associated with a worse prognosis [33]. In the molecular mutation landscape, Cluster 1 is often characterized by the absence of *IDH1* and *ATRX* mutations while frequently exhibiting mutations in genes like *EGFR*. These molecular changes serve as crucial clinical indicators of poor prognosis [34]. Notably, mutations in CIC are linked to the improved prognosis observed in Cluster 2, commonly observed in oligodendrogliomas and often linked to 1p/19q codeletion [35]. This finding is consistent with our earlier analysis of pathological subtype correlations.

Recent studies have investigated the complex associations between PTMs, molecular genetic alterations, and glioma progression. Li et al. [36] demonstrated that *IDH1* mutations induce mitochondrial hypersuccinylation, compromising mitochondrial function and conferring apoptotic resistance in glioma cells. Sturm's research revealed that *IDH1*/*2* mutations trigger D‐2‐hydroxyglutarate (2‐HG) accumulation, inhibiting α‐ketoglutarate‐dependent dioxygenases, including KDM4/6. This mechanism leads to histone hypermethylation (H3K9me3, H3K27me3), remodeling chromatin structure and driving gliomas toward a lower‐grade phenotype. The consequent epigenetic silencing may improve patient prognosis by suppressing oncogenes like PDGFRA [37]. Subsequent research expanded on PTM mechanisms in glioblastoma (GBM). Dong et al. [38] linked elevated protein arginine methyltransferase 2 (PRMT2) and asymmetric H3R8 methylation to poor GBM prognosis. Zhao et al. [39] discovered that TRIM28‐mediated SUMOylation regulates HDAC7 stability, modifying H3K27 deacetylation and activating JUN transcriptional activity. This process promotes LGALS3 secretion, inducing mesenchymal transformation of glioma stem cells and M2 macrophage polarization. Mnatsakanyan et al. [20] investigated SCD1/5's role in DNA damage repair, demonstrating its regulation of PARP1 subcellular localization. Downregulation of SCD1/5 decreases PARP1 expression, causing nuclear‐to‐cytoplasmic translocation and compromising DNA repair mechanisms. Conversely, SCD1/5 overexpression enhances PARP1 stability and DNA repair function, increasing radiotherapy and temozolomide tolerance.

Recent prognostic modeling efforts have varied in approach. Yin et al. [40] establish a prognostic model based on the Cytosine‐phosphate‐guanine (CpG) island methylation score and AUC achieving from 0.73–0.77; however, the model does not have internal validation. Karabacak et al. [41] used comprehensive clinical information (including sociodemographic, clinicopathology, diagnostic information, treatment strategy, and molecular markers) and applied five machine learning methods to establish a survival prediction model for WHO Grade 2 and 3 glioma patients, achieving AUC all above 0.8 but lacking external validation. Our study integrates molecular markers and machine learning, focusing on PTM‐related pathways. By combining multiomics data, we screened candidate genes and developed a prognostic model using advanced deep machine‐learning techniques. The primary training set from TCGA provides comprehensive transcriptome and survival data, while the CGGA cohort serves as the validation set, representing the largest multiomics glioma database in the Chinese population. Comprising about 1000 samples with over 95% Chinese participants, this approach effectively assesses the crossethnic applicability of findings originally identified in the predominantly European and American TCGA cohort. Through various algorithmic approaches, we developed and validated an 11‐gene prognostic signature for glioma. Among these genes, *TOM1L1* emerged as a critical driver of glioma progression. The suppression of *TOM1L1* significantly diminished malignant behaviors, indicating its role in promoting the aggressive phenotype of glioma. *TOM1L1* has been implicated in the progression of several cancers, such as breast cancer and clear cell renal cell carcinoma [21, 42]. It plays a critical role in the ubiquitination pathway, a key PTM mechanism regulating protein stability and signaling [43]. This interaction may explain TOM1L1's impact on glioma cell proliferation, migration, and invasion. However, the precise PTM‐related targets and pathways regulated by TOM1L1 in glioma remain unclear. Therefore, the pro‐tumorigenic mechanisms of *TOM1L1* in glioma, particularly concerning PTM pathways, warrant further investigation. Understanding how *TOM1L1* influences PTM processes like phosphorylation or ubiquitination could provide valuable insights into its role in promoting glioma progression and potentially identify new therapeutic targets.

Although some findings have been confirmed through cellular and molecular experiments, further validation in a larger clinical patient cohort is essential. Understanding the mechanistic interactions between molecules and the pathways regulating PTMs remains a critical area of investigation. To address this, we plan to validate our results using an expanded set of clinical samples. While our current study utilized established cell lines, we recognized the limitations of these models in fully recapitulating the heterogeneity of human gliomas. In future work, we plan to validate our findings using patient‐derived xenograft models, which better preserve the molecular and histological features of primary tumors. Additionally, we will conduct more extensive molecular biology experiments to elucidate how multiple PTMs work together to drive tumor progression.

## 5 Conclusions

In summary, our findings shed new light on the association between PTMs and glioma, offering a novel perspective to guide future research in this field. Different PTM expression patterns were identified as key factors influencing the prognosis of glioma patients, including aspects such as pathology, immune infiltration, and molecular mutations. Additionally, we developed a novel prognostic prediction model based on PTMs, which could assist in guiding immunotherapy for patients. These results may advance the field of precision medicine and offer new criteria for selecting treatment strategies, particularly for glioma patients with poor prognosis.

## Author Contributions

C.R. and X.J. conceived the study. Z.J., H.H., Y.G., Z.W., H.H., W.Y., H.H., L.W., and W.L. collected and analyzed data and visualized figures. Z.J. and H.H. wrote the manuscript. All authors reviewed and approved the submitted manuscript.

## Ethics Statement

The authors have nothing to report.

## Consent

The authors have nothing to report.

## Conflicts of Interest

The authors declare no conflicts of interest.

## Supporting information


**Figure S1.** The cluster analysis in glioma. (A) Tracking plot for different values of K. (B) Consensus clustering algorithm heatmap with k = 2. (C) Consensus clustering algorithm heatmap with k = 4. (D) Determination of the optimal number of clusters for PTMs clustering. (E) Bar plot of item‐consensus heatmap with k = 2, 3 and 4. (F) Bar plot of the consensus clustering algorithm with k = 2, 3 and 4. (G) The DSS curve of TCGA patients. (H) Relative expression of TOM1L1 gene in human glioma models (HS683, LN229, T98G, U251MG, U87MG) based on RNA‐seq data from the Cancer Cell Line Encyclopedia database.

**Table S1.** The list of genes involved in PTM pathways.
**Table S2.** The scores of PTM‐related prognostic genes.

## Data Availability

The data source of the manuscript is from the public database. The details were displayed in the materials and methods section.
