# Supplementary figures and images for: Unveiling the Role of Protein Posttranslational Modifications in Glioma Prognosis
Source: CNS Neurosci Ther. 2025 Mar 16;31(3):e70330. doi: 10.1111/cns.70330 (PMC11911106; doi:10.1111/cns.70330)

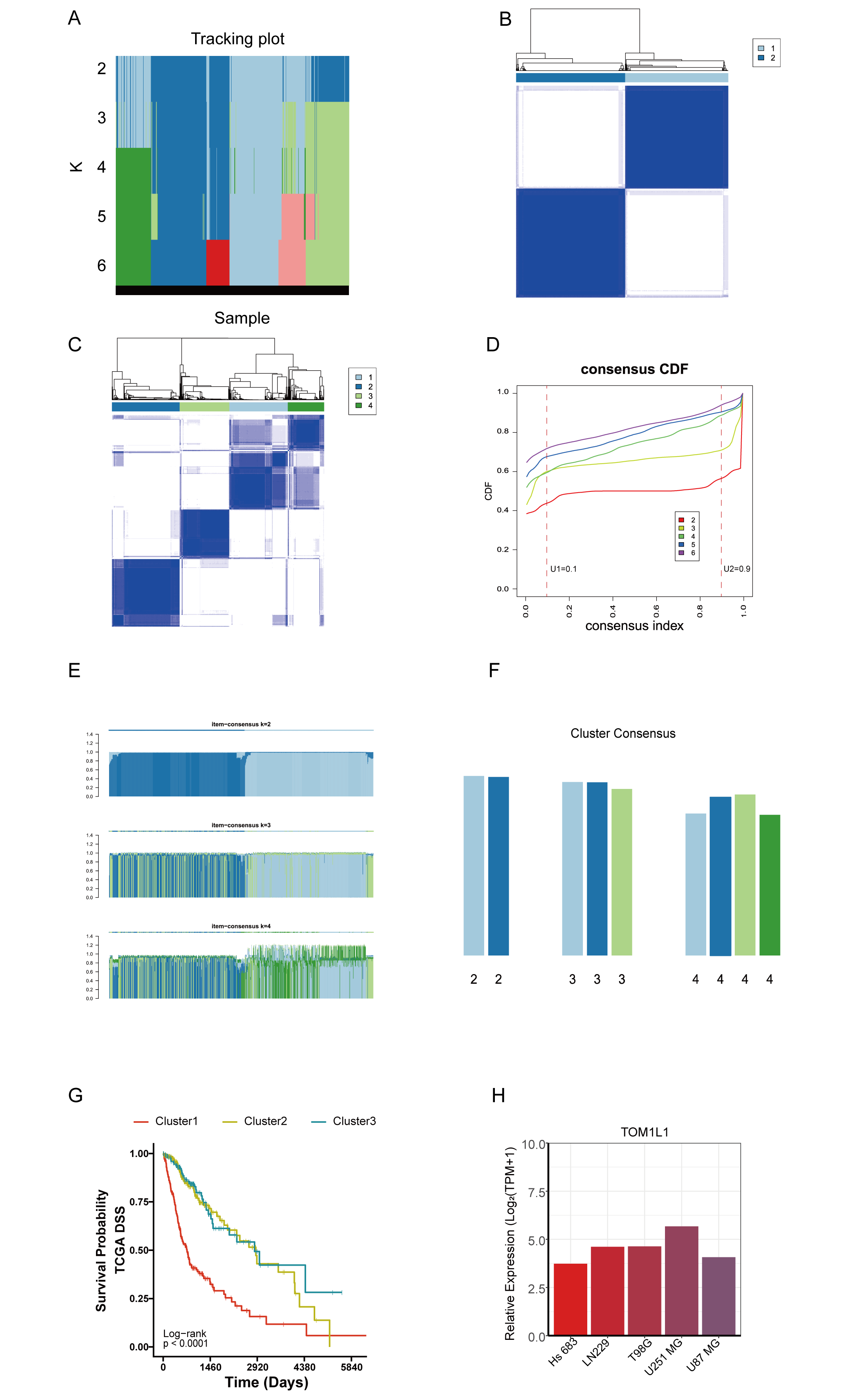

Supplement: Supplementary file 1 — Figure S1. The cluster analysis in glioma. (A) Tracking plot for different values of K. (B) Consensus clustering algorithm heatmap with k = 2. (C) Consensus clustering algorithm heatmap with k = 4. (D) Determination of the optimal number of clusters for PTMs clustering. (E) Bar plot of item‐consensus heatmap with k = 2, 3 and 4. (F) Bar plot of the consensus clustering algorithm with k = 2, 3 and 4. (G) The DSS curve of TCGA patients. (H) Relative expression of TOM1L1 gene in human glioma models (HS683, LN229, T98G, U251MG, U87MG) based on RNA‐seq data from the Cancer Cell Line Encyclopedia database. [file CNS-31-e70330-s001.tif]
