# Supplementary material for: Unveiling the Role of Protein Posttranslational Modifications in Glioma Prognosis
Source: CNS Neurosci Ther. 2025 Mar 16;31(3):e70330. doi: 10.1111/cns.70330 (PMC11911106; doi:10.1111/cns.70330)
Supplement: Supplementary file 2 — Table S1. The list of genes involved in PTM pathways. Table S2. The scores of PTM‐related prognostic genes. [file CNS-31-e70330-s002.docx]

Supplement Table1 The list of genes involved in PTMs pathways

| Term | Gene |
| --- | --- |
| Phosphorylation | AAK1, AATK, ABL1, ABL2, ACE, ACP1, ACP3, ACP4, ACVR1, ACVR1B, ACVR1C, ACVR2A, ACVR2B, ACVRL1, ADAR, ADARB1, ADCK1, ADCK2, ADCK5, ADCY1, ADIPOQ, AKAP9, AKT1, AKT2, AKT3, ALK, ALPK1, ALPK2, ALPK3, AMHR2, ANK2, ANKK1, ARAF, ATM, ATP13A2, ATP23, ATR, AURKA, AURKB, AURKC, AVP, AXL, BAD, BAX, BAZ1B, BCKDK, BCL2, BCR, BLK, BMP2K, BMPR1A, BMPR1B, BMPR2, BMX, BRAF, BRD2, BRD4, BRSK1, BRSK2, BTK, BUB1, BUB1B, C8orf44-SGK3, CAD, CALM1, CALM2, CALM3, CAMK1, CAMK1D, CAMK1G, CAMK2A, CAMK2B, CAMK2D, CAMK2G, CAMK4, CAMKK1, CAMKK2, CAMKV, CASK, CAV1, CCL2, CCL3, CCL5, CCL8, CCNB1, CCND1, CCND3, CCNE1, CCNK, CD247, CD3D, CD3E, CD3G, CD4, CDC14A, CDC14B, CDC14C, CDC25A, CDC25B, CDC25C, CDC42BPA, CDC42BPB, CDC42BPG, CDC7, CDK1, CDK10, CDK11A, CDK11B, CDK12, CDK13, CDK14, CDK15, CDK16, CDK17, CDK18, CDK19, CDK2, CDK20, CDK3, CDK4, CDK5, CDK5R1, CDK5RAP3, CDK6, CDK7, CDK8, CDK9, CDKL1, CDKL2, CDKL3, CDKL4, CDKL5, CDKN1A, CDKN1B, CDKN3, CHEK1, CHEK2, CHKA, CHP1, CHUK, CIITA, CILK1, CIT, CKS1B, CLK1, CLK2, CLK3, CLK4, COQ8A, COQ8B, CORO1C, CPNE3, CPPED1, CREB1, CREBBP, CRIM1, CSF1R, CSF2RB, CSK, CSNK1A1, CSNK1A1L, CSNK1D, CSNK1E, CSNK1G1, CSNK1G2, CSNK1G3, CSNK2A1, CSNK2A2, CSNK2B, CTDNEP1, CTDP1, CTDSP1, CTDSP2, CTDSPL, CTDSPL2, CUL1, DAPK1, DAPK2, DAPK3, DCAF1, DCLK1, DCLK2, DCLK3, DCY1, DDR1, DDR2, DDX3X, DLG1, DMPK, DNAJC6, DSTYK, DUSP1, DUSP10, DUSP11, DUSP12, DUSP13, DUSP14, DUSP15, DUSP16, DUSP18, DUSP19, DUSP2, DUSP21, DUSP22, DUSP23, DUSP26, DUSP28, DUSP29, DUSP3, DUSP4, DUSP5, DUSP6, DUSP7, DUSP8, DUSP9, DYRK1A, DYRK1B, DYRK2, DYRK3, DYRK4, E2F1, EEF2K, EFEMP1, EFNA3, EFNA4, EFNB3, EGFR, EIF2AK1, EIF2AK2, EIF2AK3, EIF2AK4, ENG, ENPP1, LOC102724428, EPHA1, EPHA10, EPHA2, EPHA3, EPHA4, EPHA5, EPHA6, EPHA7, EPHA8, EPHB1, EPHB2, EPHB3, EPHB4, EPHB6, ERBB2, ERBB3, ERBB4, ERN1, ERN2, ERRFI1, EYA1, EYA2, EYA3, EYA4, FAM20A, FAM20C, FASTK, FER, FES, FGFR1, FGFR2, FGFR3, FGFR4, FGFRL1, FGR, FIG4, FLT1, FLT3, FLT4, FRK, FYN, GAK, GPNMB, GRB2, GREM1, GRK1, GRK2, GRK3, GRK4, GRK5, GRK6, GRK7, GSK3A, GSK3B, GUCY2C, GUCY2D, GUCY2F, HACD2, HASPIN, HCK, HIPK1, HIPK2, HIPK3, HIPK4, HJV, HLA-DRA, HLA-DRB1, HLA-DRB3, HLA-DRB4, HLA-DRB5, HRAS, HSPB8, HTATIP2, HUNK, IGF1, IGF1R, IGF2R, IKBKB, IKBKE, IL3, IL3RA, ILK, ILKAP, IMPACT, INS, INSR, INSRR, IRAK1, IRAK2, IRAK3, IRAK4, IRS1, ITK, ITPKA, JAK1, JAK2, JAK3, KALRN, KDR, KIT, KITLG, KSR1, KSR2, LATS1, LATS2, LCK, LIMK1, LIMK2, LMTK2, LMTK3, LRRK1, LRRK2, LTBP1, LTBP4, LTK, LYN, MAK, MAP2, MAP2K1, MAP2K2, MAP2K3, MAP2K4, MAP2K5, MAP2K6, MAP2K7, MAP3K1, MAP3K10, MAP3K11, MAP3K12, MAP3K13, MAP3K14, MAP3K15, MAP3K19, MAP3K2, MAP3K20, MAP3K21, MAP3K3, MAP3K4, MAP3K5, MAP3K6, MAP3K7, MAP3K8, MAP3K9, MAP4K1, MAP4K2, MAP4K3, MAP4K4, MAP4K5, MAPK1, MAPK10, MAPK11, MAPK12, MAPK13, MAPK14, MAPK15, MAPK3, MAPK4, MAPK6, MAPK7, MAPK8, MAPK9, MAPKAPK2, MAPKAPK3, MAPKAPK5, MARK1, MARK2, MARK3, MARK4, MAST1, MAST2, MAST3, MAST4, MASTL, MATK, MDP1, MELK, MERTK, MET, MEX3B, MINK1, MINPP1, MKNK1, MKNK2, MLKL, MMD, MMD2, MOB1B, MOK, MOS, MRE11, MST1R, MTM1, MTMR14, MTMR2, MTMR3, MTMR4, MTMR6, MTMR7, MTOR, MUSK, MVP, MYH3, MYH6, MYH8, MYLK, MYLK2, MYLK3, MYLK4, MYO3A, MYO3B, NBN, NEDD8, NEK1, NEK10, NEK11, NEK2, NEK3, NEK4, NEK5, NEK6, NEK7, NEK8, NEK9, NIM1K, NLK, NLRP12, NME2, NPM1, NPR1, NPR2, NRBP1, NRBP2, NRK, NRP1, NRP2, NTRK1, NTRK2, NTRK3, NUAK1, NUAK2, NUP85, OBSCN, OXSR1, PAK1, PAK2, PAK3, PAK4, PAK5, PAK6, PALD1, PAN3, PASK, PBK, PCK1, PCNT, PDGFA, PDGFB, PDGFC, PDGFD, PDGFRA, PDGFRB, PDGFRL, PDIK1L, PDK1, PDK2, PDK3, PDK4, PDP1, PDP2, PDPK1, PDXP, PEAK1, PEAK3, PGAM5, PGP, PHKA1, PHKA2, PHKG1, PHKG2, PHLPP1, PHLPP2, PHPT1, PIK3C3, PIK3CA, PIK3CB, PIK3CG, PIK3R1, PIK3R4, PIKFYVE, PIM1, PIM2, PIM3, PINK1, PKDCC, PKM, PKMYT1, PKN1, PKN2, PKN3, PLCG2, PLK1, PLK2, PLK3, PLK4, PLK5, PNCK, POMK, PP2D1, PPA2, PPEF1, PPEF2, PPM1A, PPM1B, PPM1D, PPM1E, PPM1F, PPM1G, PPM1H, PPM1J, PPM1K, PPM1L, PPM1M, PPM1N, PPP1CA, PPP1CB, PPP1CC, PPP1R3B, PPP1R3C, PPP1R3D, PPP1R3E, PPP1R9B, PPP2CA, PPP2CB, PPP2R1A, PPP2R5A, PPP2R5B, PPP2R5D, PPP3CA, PPP3CB, PPP3CC, PPP3R1, PPP4C, PPP4R1, PPP5C, PPP6C, PPTC7, PRAG1, PRKAA1, PRKAA2, PRKAB1, PRKAB2, PRKACA, PRKACB, PRKACG, PRKAG1, PRKAG2, PRKAG3, PRKAR1A, PRKAR1B, PRKAR2A, PRKAR2B, PRKCA, PRKCB, PRKCD, PRKCE, PRKCG, PRKCH, PRKCI, PRKCQ, PRKCZ, PRKD1, PRKD2, PRKD3, PRKDC, PRKG1, PRKG2, PRKX, PRKY, PRLR, PRPF4B, PSKH1, PSKH2, PTEN, PTK2, PTK2B, PTK6, PTK7, PTP4A1, PTP4A2, PTP4A3, PTPA, PTPDC1, PTPMT1, PTPN1, PTPN11, PTPN12, PTPN13, PTPN14, PTPN18, PTPN2, PTPN20, PTPN21, PTPN22, PTPN23, PTPN3, PTPN4, PTPN5, PTPN6, PTPN7, PTPN9, PTPRA, PTPRB, PTPRC, PTPRD, PTPRE, PTPRF, PTPRG, PTPRH, PTPRJ, PTPRK, PTPRM, PTPRN, PTPRN2, PTPRO, PTPRQ, PTPRR, PTPRS, PTPRT, PTPRU, PTPRZ1, PXK, RAD50, RAF1, RAP2A, RAP2B, RAP2C, RASSF2, RB1, RBX1, RET, RHOA, RIOK1, RIOK2, RIOK3, RIPK1, RIPK2, RIPK3, RIPK4, RNASEL, RNGTT, ROCK1, ROCK2, ROR1, ROR2, ROS1, RPAP2, RPS6KA1, RPS6KA2, RPS6KA3, RPS6KA4, RPS6KA5, RPS6KA6, RPS6KB1, RPS6KB2, RPS6KC1, RPS6KL1, RSKR, RYK, SBF1, SBK1, SBK2, SBK3, SCYL1, SCYL2, SGK1, SGK2, SGK3, SHC1, SIK1, SIK2, SIK3, SKP1, SKP2, SLK, SMG1, SNRK, SOS1, SOSTDC1, SPEG, SQSTM1, SRC, SRMS, SRPK1, SRPK2, SRPK3, SSH1, SSH2, SSH3, SSU72, SSU72L1, SSU72L2, SSU72L3, SSU72L4, SSU72L5, SSU72L6, STK10, STK11, STK16, STK17A, STK17B, STK19, STK24, STK25, STK26, STK3, STK31, STK32A, STK32B, STK32C, STK33, STK35, STK36, STK38, STK38L, STK39, STK4, STK40, STKLD1, STRADA, STRADB, STYK1, STYX, STYXL1, STYXL2, SYK, TAB1, TAF1, TAF1L, TAOK1, TAOK2, TAOK3, TBCK, TBK1, TEC, TEK, TESK1, TESK2, TEX14, TFDP1, TGFBR1, TGFBR2, TGFBR3, TGFBR3L, THAP7, TIE1, TIMM50, TLK1, TLK2, TNIK, TNK1, TNK2, TNKS1BP1, TNNI3K, TNS1, TNS2, TNS3, TOM1L1, TOP1, TP53RK, TPTE, TRIB1, TRIB2, TRIM24, TRIM28, TRIO, TRPM6, TRPM7, TSSK1B, TSSK2, TSSK3, TSSK4, TSSK6, TTBK1, TTBK2, TTK, TTN, TWF1, TXK, TYK2, TYRO3, UBASH3B, UBE2M, UBLCP1, UHMK1, ULK1, ULK2, ULK3, ULK4, VAV1, VEGFA, VEGFC, VPS16, VRK1, VRK2, VRK3, WEE1, WEE2, WNK1, WNK2, WNK3, WNK4, YES1, YWHAH, ZAP70 |
| Disulfide formation | CALR, CANX, CCS, CHCHD4, CRELD1, CRELD2, DNAJC10, DNAJC3, ENOX1, ERO1A, ERO1B, ERP27, ERP29, ERP44, GFER, GLRX, GLRX2, GSR, GSTO1, GSTO2, HSP90B1, HSPA5, ITGB3, NXN, P4HB, PDIA2, PDIA3, PDIA4, PDIA5, PDIA6, PDILT, PGK1, PIGK, QSOX1, QSOX2, RRM1, RRM2, RRM2B, SCO2, STAB1, STAB2, TMX1, TMX2, TMX3, TMX4, TXN, TXN2, TXNDC12, TXNDC17, TXNDC5, TXNDC8, TXNL1, VAPA, VKORC1, VKORC1L1 |
| Palmitoylation | ABHD10, ABHD12, ABHD13, ABHD17A, ABHD17B, ABHD17C, CLIP3, CPT1A, CPT1B, CPT1C, CPT2, GLUL, GOLGA7, GOLGA7B, HHAT, HHATL, LRAT, LYPLA1, LYPLA2, LYPLAL1, PORCN, PPT1, PPT2, SELENOK, SPTLC1, SPTLC2, SPTLC3, SPTSSA, SPTSSB, YKT6, ZDHHC1, ZDHHC11, ZDHHC11B, ZDHHC12, ZDHHC13, ZDHHC14, ZDHHC15, ZDHHC16, ZDHHC17, ZDHHC18, ZDHHC19, ZDHHC2, ZDHHC20, ZDHHC21, ZDHHC22, ZDHHC23, ZDHHC24, ZDHHC3, ZDHHC4, ZDHHC5, ZDHHC6, ZDHHC7, ZDHHC8, ZDHHC9 |
| Citrullination | PADI1, PADI2, PADI3, PADI4, PADI6 |
| Methylation | ALKBH4, ANTKMT, ASH1L, ASH2L, ATPSCKMT, ATRX, AUTS2, BAZ2A, BHMT, BOD1, BOD1L1, BPTF, CALM1, CAMKMT, CARM1, CBX1, CBX2, CBX3, CBX4, CBX5, CBX6, CBX8, CDY1, CDY1B, CDYL, CDYL2, CHD1, CHD5, CHD8, CSKMT, CXXC1, DOT1L, DPF2, DPPA3, DPY30, DYDC1, DYDC2, EED, EEF1A1, EEF1AKMT1, EEF1AKMT2, EEF1AKMT3, EEF2, EEF2KMT, EHMT1, EHMT2, ETF1, ETFB, ETFBKMT, EZH1, EZH2, FAM156A, FAM156B, FAM86B1, FAM86B2, FAM98A, FAM98B, FBL, FBLL1, FBXL19, FMR1, GCG, GLYR1, GSPT1, HCFC1, HCFC2, HDGFL2, HSPA8, ICMT, ING1, ING2, ING3, ING4, ING5, JARID2, JMJD7, KANSL1, KANSL2, KANSL3, KAT8, KDM1A, KDM2A, KDM2B, KDM4A, KDM4C, KDM5A, KDM6A, KDM6B, KDM7A, KDM8, KIN, KMT2A, KMT2B, KMT2C, KMT2D, KMT2E, KMT5A, KMT5B, KMT5C, L3MBTL1, L3MBTL2, L3MBTL3, LCMT1, LRWD1, MBTD1, MCRS1, MECOM, MECP2, METTL18, METTL21A, METTL21C, METTL21EP, METTL22, METTL23, MORC3, MORC4, MPHOSPH8, MSH6, MSL3, MTF2, N6AMT1, NCAPD3, NCAPG2, NDUFAF7, NELFA, NELFE, NSD1, NSD2, NSD3, NTMT1, NTMT2, OGT, PCMT1, PHF1, PHF13, PHF19, PHF2, PHF20, PHF20L1, PHF8, PPME1, PRDM12, PRDM13, PRDM14, PRDM16, PRDM2, PRDM6, PRDM7, PRDM8, PRDM9, PRMT1, PRMT2, PRMT3, PRMT5, PRMT6, PRMT7, PRMT8, PWP1, PWWP2A, PYGO1, RAB6A, RAG2, RBBP5, RPS2, RRP8, SETBP1, SETD1A, SETD1B, SETD2, SETD3, SETD4, SETD5, SETD6, SETD7, SETDB1, SETDB2, SETMAR, SGF29, SMARCA5, SMYD1, SMYD2, SMYD3, SMYD5, SNRPB, SNRPD3, SPIN1, SPIN2A, SPIN2B, SPIN3, SPIN4, SUV39H1, SUV39H2, SUZ12, TAF1, TAF7, TDRD3, THAP7, TP53BP1, TRIM24, TRMT112, TTLL12, UHRF1, UTY, VCP, VCPKMT, WDR5, WDR5B, WDR82, ZCWPW1, ZCWPW2, ZMYND11, ZMYND8, ZZEF1, ZZZ3 |
| ADP-ribosylation | ADPRH, ADPRS, APLF, ARAP1, ARAP2, ARF1, ARFGAP1, ARFGAP3, ARFGEF1, ARFGEF2, ASAP1, ASAP2, BANF1, CGAS, CHD1L, CLTB, COPA, CYTH1, CYTH2, CYTH3, CYTH4, GBF1, GPLD1, HPF1, KAT2B, MACROD1, MACROD2, MACROH2A1, MARCHF6-DT, OARD1, PARP1, PARP10, PARP11, PARP12, PARP14, PARP15, PARP16, PARP2, PARP3, PARP6, PARP8, PARP9, PNKP, PUM3, SPINDOC, TINF2, TNKS, TNKS2, XRCC1, ZC3HAV1 |
| Acetylation | AANAT, ABHD14B, ACTB, ACTBL2, ACTG1, ACTL6A, ACTL6B, ACTL9, ARID5A, ARRB1, ATAT1, ATF2, AUTS2, BAG6, BAZ1A, BAZ1B, BAZ2A, BEX4, BLOC1S1, BMAL1, BRCA1, BRCA2, BRD1, BRD8, BRMS1, BRPF1, BRPF3, CCAR2, CDY1, CDY1B, CDY2A, CDY2B, CEP295, CLOCK, CPA4, CREBBP, DDX21, DDX3X, DEK, DIP2A, DIP2B, DMAP1, DR1, DSCC1, DYRK1A, EP300, EP400, EPC1, EPC2, ERCC6, ESCO1, ESCO2, FAM161A, FLCN, FLNA, FNTA, FOXO1, FRY, GATA2, GSK3B, GTF2B, GTF3C4, HAT1, HCFC1, HDAC1, HDAC10, HDAC11, HDAC2, HDAC3, HDAC4, HDAC6, HDAC7, HDAC8, HDAC9, HINT2, IFNG, ING3, ING4, ING5, JADE1, JADE2, KANSL1, KANSL1L, KANSL2, KANSL3, KAT14, KAT2A, KAT2B, KAT5, KAT6A, KAT6B, KAT7, KAT8, KLF15, LACRT, MAGEA2, MAGEA2B, MAP3K7, MAPT, MBIP, MBTD1, MCM3AP, MCRS1, MEAF6, MIER1, MIER2, MIR182, MIR217, MIR34A, MORF4L1, MORF4L2, MRGBP, MSL1, MSL2, MSL3, MSL3P1, MTA1, MTA2, MTA3, MYBBP1A, NAA10, NAA11, NAA15, NAA16, NAA20, NAA25, NAA30, NAA35, NAA40, NAA50, NAA60, NAA80, NAT10, NAT8, NAT8B, NCOA1, NCOA3, NDN, NEK3, NFYA, NFYB, NFYC, NNMT, OGT, PARK7, PHF10, PHF20, PHF20L1, PML, POLE3, POLE4, POTEE, POTEF, POTEI, POTEJ, POTEKP, PPARGC1A, PRKAA1, PRKAA2, RBBP4, RBBP7, RRP8, RUVBL1, RUVBL2, SET, SF3B1, SGF29, SIRT1, SIRT2, SIRT3, SIRT4, SIRT5, SIRT6, SIRT7, SMARCA5, SOX4, SPHK1, SPRED1, SPRED2, SRCAP, SUPT3H, SUV39H1, TADA2A, TADA2B, TADA3, TAF1, TAF10, TAF1L, TAF5L, TAF9, TAOK1, TPPP, TRRAP, USP22, VPS72, WDR5, YEATS2, YEATS4, ZZZ3 |
| Ubiquitination | ABL1, ABRAXAS1, ABRAXAS2, ABTB1, ADGRB1, AIMP2, AIRE, AKT1, ALG13, AMBRA1, AMER1, AMFR, AMN1, ANAPC1, ANAPC10, ANAPC11, ANAPC13, ANAPC15, ANAPC16, ANAPC2, ANAPC4, ANAPC5, ANAPC7, ANGPT1, ANKIB1, ANKRD9, ANKZF1, APPBP2, AREL1, ARIH1, ARIH2, ARMC8, ARRB1, ARRB2, ARRDC1, ARRDC3, ARRDC4, ASB1, ASB10, ASB11, ASB12, ASB13, ASB14, ASB15, ASB16, ASB17, ASB18, ASB2, ASB3, ASB4, ASB5, ASB6, ASB7, ASB8, ASB9, ASCC2, ASCC3, ASNS, ATG3, ATG5, ATG7, ATM, ATXN3, ATXN3L, AUP1, AXIN1, BABAM2, BAG2, BAG5, BAG6, BAP1, BARD1, BCAP31, BCL10, BCL2, BCOR, BEX1, BEX2, BEX3, BEX4, BFAR, BIRC2, BIRC3, BIRC6, BIRC7, BIRC8, BLMH, BMI1, BRAP, BRCA1, BRCA2, BRCC3, BTBD1, BTBD6, BTRC, BUB1B, BUB3, C10orf90, CACYBP, CAD, CALR, CALR3, CAMLG, CAND1, CANX, CASK, CASP1, CASP8, CASP8AP2, CAV1, CBFB, CBL, CBLB, CBLC, CBLL1, CBLL2, CBX2, CBX4, CBX6, CBX7, CBX8, CCDC22, CCDC47, CCNE1, CCNF, CDC14B, CDC16, CDC20, CDC20B, CDC23, CDC26, CDC27, CDC34, CDC73, CDK5, CDK5RAP3, CDKN1B, CENPS, CENPX, CEP63, CHFR, CHP1, CKS1B, CKS2, CLGN, CLSPN, CNOT4, COMMD1, COP1, COP1P1, COPS4, COPS5, COPS6, CRBN, CRY1, CTNNB1, CTPS1, CTR9, CUEDC1, CUEDC2, CUL1, CUL2, CUL3, CUL4A, CUL4B, CUL5, CUL7, CUL9, CXCR4, CYLD, DAW1, DAXX, DCAF1, DCAF10, DCAF11, DCAF12, DCAF12L1, DCAF12L2, DCAF13, DCAF15, DCAF16, DCAF17, DCAF4, DCAF4L1, DCAF4L2, DCAF5, DCAF6, DCAF7, DCAF8, DCAF8L1, DCAF8L2, DCUN1D1, DCUN1D2, DCUN1D3, DCUN1D4, DCUN1D5, DDA1, DDB1, DDB2, DDI2, DDX3X, DERL1, DERL2, DERL3, DESI1, DESI2, DET1, DHX16, DMAC2, DNAAF10, DNAJA1, DNAJB2, DNAJB9, DNAJC10, DTL, DTX3L, DYRK2, DZIP3, ECPAS, EDEM1, EDEM2, EDEM3, EIF3F, EIF3H, ELOB, ELOC, ENC1, ENTREP1, ERCC8, ERLEC1, ERLIN1, ERLIN2, FAAP20, FAF1, FAF2, FAM107A, FAM8A1, FANCI, FANCL, FANCM, FBH1, FBXL12, FBXL13, FBXL14, FBXL15, FBXL16, FBXL17, FBXL18, FBXL19, FBXL2, FBXL20, FBXL21P, FBXL22, FBXL3, FBXL4, FBXL5, FBXL6, FBXL7, FBXL8, FBXO10, FBXO11, FBXO15, FBXO17, FBXO2, FBXO21, FBXO22, FBXO24, FBXO25, FBXO27, FBXO28, FBXO3, FBXO30, FBXO31, FBXO32, FBXO33, FBXO38, FBXO39, FBXO4, FBXO40, FBXO41, FBXO42, FBXO44, FBXO45, FBXO48, FBXO5, FBXO6, FBXO7, FBXO8, FBXO9, FBXW10, FBXW11, FBXW12, FBXW2, FBXW4, FBXW5, FBXW7, FBXW8, FBXW9, FEM1A, FEM1B, FEM1C, FOXF2, FOXL2, FOXRED2, FYN, FZD4, FZD5, FZD6, FZD8, FZR1, GABARAP, GAN, GART, GATB, GCLC, GCLM, GCNA, GGA1, GGA2, GGA3, GID4, GID8, GLMN, GMCL2, GMPS, GNL3L, GPR37, GPS2, GRIK2, GSK3A, GSK3B, GSS, GTPBP4, H2BC1, H2BC10, H2BC11, H2BC12, H2BC13, H2BC14, H2BC15, H2BC17, H2BC3, H2BC4, H2BC5, H2BC6, H2BC7, H2BC8, H2BC9, HABP4, HACE1, HDAC3, HDAC6, HDAC8, HECTD1, HECTD2, HECTD3, HECW2, HERC1, HERC2, HERC3, HERC4, HERC5, HERC6, HERPUD1, HGS, HINT1, HLA-A, HLTF, HSP90AA1, HSP90AB1, HSP90B1, HSPA14, HSPA1A, HSPA1B, HSPA1L, HSPA2, HSPA4, HSPA5, HSPA6, HSPA8, HSPA9, HSPB1, HSPBP1, HUWE1, IDE, IFI27, IKBKG, ILRUN, INAVA, IRF2BP1, ISG15, ITCH, IVNS1ABP, JARID2, JKAMP, JOSD1, JOSD2, KBTBD13, KBTBD6, KBTBD7, KBTBD8, KCNH2, KCTD10, KCTD13, KCTD17, KCTD2, KCTD21, KCTD5, KCTD6, KCTD7, KCTD9, KDM1A, KEAP1, KLHDC1, KLHDC10, KLHDC2, KLHDC3, KLHL11, KLHL12, KLHL13, KLHL15, KLHL2, KLHL20, KLHL21, KLHL22, KLHL24, KLHL25, KLHL3, KLHL36, KLHL40, KLHL41, KLHL42, KLHL5, KLHL7, KLHL8, KLHL9, LAPTM5, LEO1, LGR4, LGR5, LGR6, LIMK1, LMO7, LNPEP, LNX1, LONRF1, LRP5, LRP6, LRR1, LRRC41, LRRK2, LRSAM1, LTN1, LZTR1, MAD2L1, MAD2L2, MAEA, MAGEA2, MAGEA2B, MAGEC2, MAGEL2, MALT1, MAN1A1, MAN1A2, MAN1B1, MAN1C1, MAP3K1, MAP3K7, MAPK9, MARCHF1, MARCHF5, MARCHF6, MARCHF6-DT, MARCHF7, MARCHF8, MARK4, MAVS, MDM2, MED1, MED10, MED11, MED12, MED17, MED18, MED21, MED27, MED30, MED31, MED6, MED7, MED8, MEX3C, MGRN1, MIB2, MID1, MINAR1, MINDY1, MINDY2, MINDY3, MINDY4, MINDY4B, MIR101-1, MIR138-1, MKLN1, MKRN1, MKRN3, MOCS3, MTA1, MTBP, MTHFD1, MTHFS, MUL1, MVB12A, MVB12B, MYCBP2, MYLIP, MYSM1, N4BP1, N4BP2, NAE1, NBR1, NCCRP1, NDFIP1, NDFIP2, NEDD4, NEDD4L, NEMF, NEURL1, NFE2L2, NFX1, NHLRC1, NHLRC3, NMI, NOD1, NOD2, NOP53, NPEPPS, NPLOC4, NPM1, NSFL1C, NSMCE3, NUP62, NXN, OBI1, OGT, OS9, OTUB1, OTUB2, OTUD1, OTUD3, OTUD4, OTUD5, OTUD6A, OTUD6B, OTUD7A, OTUD7B, OTULIN, OTULINL, PAF1, PAICS, PAN2, PARK2, PARK7, PARP10, PAXIP1, PCCB, PCGF1, PCGF2, PCGF3, PCGF5, PCGF6, PCMTD1, PCNA, PCNP, PDCD6, PEF1, PELI1, PELI2, PELI3, PELP1, PER2, PEX10, PEX12, PEX13, PEX14, PEX2, PEX5, PHC1, PHC2, PHC3, PHF23, PIAS1, PIAS2, PIAS3, PIAS4, PIN1, PINK1, PINX1, PJA1, PJA2, PLAA, PLK1, PML, PPARA, PPIA, PPIL2, PRAME, PRAMEF6, PRAMEF9, PRICKLE1, PRKCE, PRKCG, PRKDC, PRKN, PRMT3, PRPF19, PRPF8, PRPS2, PSMA1, PSMA2, PSMA3, PSMA4, PSMA5, PSMA6, PSMA7, PSMA8, PSMB1, PSMB10, PSMB11, PSMB2, PSMB3, PSMB4, PSMB5, PSMB6, PSMB7, PSMB8, PSMB9, PSMC1, PSMC2, PSMC3, PSMC4, PSMC5, PSMC6, PSMD1, PSMD10, PSMD11, PSMD12, PSMD13, PSMD14, PSMD2, PSMD3, PSMD4, PSMD5, PSMD6, PSMD7, PSMD8, PSMD9, PSME1, PSME2, PSME3, PSME4, PSMF1, PTEN, PTPN22, PTTG1IP, RAD18, RAD23A, RAD23B, RAD51, RAE1, RAG1, RANBP10, RANBP9, RASSF5, RBBP6, RBCK1, RBX1, RC3H1, RC3H2, RCHY1, RFFL, RFPL1, RFPL2, RFPL3, RFPL4A, RFPL4AL1, RHBDD2, RHOBTB2, RING1, RIPK2, RLIM, RMND5A, RMND5B, RNF10, RNF103, RNF11, RNF111, RNF112, RNF114, RNF115, RNF121, RNF122, RNF123, RNF125, RNF126, RNF13, RNF130, RNF135, RNF138, RNF139, RNF14, RNF141, RNF144A, RNF144B, RNF146, RNF152, RNF166, RNF167, RNF168, RNF175, RNF180, RNF181, RNF182, RNF183, RNF185, RNF186, RNF187, RNF19A, RNF19B, RNF2, RNF20, RNF208, RNF213, RNF216, RNF217, RNF220, RNF25, RNF26, RNF31, RNF34, RNF4, RNF40, RNF41, RNF43, RNF5, RNF6, RNF7, RNF8, RNFT1, RPL11, RPL23, RPL37, RPL5, RPS15, RPS2, RPS20, RPS27A, RPS27AP11, RPS3, RPS7, RRAGA, RSPO1, RSPO2, RSPO3, RSPO4, RTF1, RUSC1, SAE1, SASH1, SEC61B, SEL1L, SELENOS, SEM1, SENP1, SENP2, SENP3, SENP5, SENP6, SENP7, SENP8, SEPTIN4, SEPTIN5, SERBP1, SGTA, SH3BGRL, SH3RF1, SH3RF2, SH3RF3, SHARPIN, SHMT2, SHPRH, SIAH1, SIAH2, SIAH3, SIMC1, SIRT2, SIRT7, SKIC8, SKP1, SKP1P2, SKP2, SMAD3, SMAD7, SMARCAD1, SMURF1, SMURF2, SNCA, SNCAIP, SOBP, SOCS1, SOCS3, SOX4, SPATA2, SPHK1, SPOP, SPOPL, SPRTN, SPRY2, SPSB1, SPSB2, SPSB3, SPSB4, SQSTM1, STAM, STAM2, STAMBP, STAMBPL1, STT3B, STUB1, SUGT1, SUV39H2, SVBP, SVIP, SYVN1, TAB2, TAB3, TAF1, TANK, TBC1D7, TCF25, TDG, TGFBR1, THOP1, TICAM1, TMEM129, TMEM183A, TMEM183BP, TMEM67, TMUB1, TMUB2, TNFAIP1, TNFAIP3, TNIP1, TNKS, TNKS2, TOLLIP, TOM1, TOM1L1, TOM1L2, TOP2A, TOPORS, TP53, TP53INP2, TPP2, TRAF1, TRAF2, TRAF3, TRAF3IP2, TRAF5, TRAF6, TRAF7, TRAIP, TRIB1, TRIB2, TRIB3, TRIM11, TRIM13, TRIM17, TRIM2, TRIM21, TRIM22, TRIM23, TRIM25, TRIM27, TRIM3, TRIM31, TRIM32, TRIM34, TRIM36, TRIM37, TRIM38, TRIM39, TRIM4, TRIM41, TRIM44, TRIM5, TRIM50, TRIM52, TRIM56, TRIM58, TRIM6, TRIM62, TRIM63, TRIM68, TRIM69, TRIM71, TRIM72, TRIM9, TRIP12, TRIP4, TRPC4AP, TSC1, TSG101, TSPAN17, TSPO, TSPYL5, TTC3, TTC36, TUBA1A, TUBA1B, TUBA1C, TUBA3C, TUBA3D, TUBA3E, TUBA4A, TUBA4B, TUBA8, TUBAL3, TUBB, TUBB1, TUBB2A, TUBB2B, TUBB3, TUBB4A, TUBB4B, TUBB6, TUBB8, U2AF2, UBA1, UBA2, UBA3, UBA5, UBA52, UBA6, UBA7, UBAC1, UBAP1, UBAP1L, UBB, UBC, UBE2A, UBE2B, UBE2C, UBE2D1, UBE2D2, UBE2D3, UBE2D4, UBE2E1, UBE2E2, UBE2E3, UBE2F, UBE2G1, UBE2G2, UBE2H, UBE2I, UBE2J1, UBE2J2, UBE2K, UBE2L3, UBE2L5, UBE2L6, UBE2M, UBE2N, UBE2NL, UBE2O, UBE2Q1, UBE2Q2, UBE2QL1, UBE2R2, UBE2S, UBE2T, UBE2U, UBE2V1, UBE2V2, UBE2W, UBE2Z, UBE3A, UBE3B, UBE3C, UBE3D, UBE4A, UBE4B, UBOX5, UBQLN1, UBQLN2, UBQLN3, UBR1, UBR2, UBR3, UBR4, UBR5, UBXN1, UBXN10, UBXN11, UBXN2A, UBXN2B, UBXN4, UBXN7, UBXN8, UCHL1, UCHL3, UCHL5, UEVLD, UFD1, UFL1, UFSP2, UHRF1, UHRF2, UMOD, UNKL, USP1, USP10, USP11, USP12, USP13, USP14, USP15, USP16, USP17L1, USP17L10, USP17L11, USP17L12, USP17L13, USP17L15, USP17L17, USP17L18, USP17L19, USP17L2, USP17L20, USP17L21, USP17L22, USP17L23, USP17L24, USP17L3, USP17L4, USP17L5, USP17L6P, USP17L7, USP17L8, USP18, USP19, USP2, USP20, USP21, USP22, USP24, USP25, USP26, USP27X, USP28, USP29, USP3, USP30, USP31, USP32, USP33, USP34, USP35, USP36, USP37, USP38, USP39, USP4, USP40, USP42, USP43, USP44, USP45, USP46, USP47, USP48, USP49, USP5, USP50, USP51, USP53, USP54, USP6, USP7, USP8, USP9X, USP9Y, USPL1, VCP, VCPIP1, VHL, VPS28, VPS36, VPS54, WAC, WASHC1, WBP1L, WDR24, WDR26, WDR48, WDR77, WDTC1, WFS1, WNK1, WNT3A, WSB1, WSB2, WWP1, WWP2, XIAP, YOD1, YPEL5, ZBTB16, ZC3H12A, ZC4H2, ZER1, ZFAND2B, ZFP91, ZMYM2, ZNF598, ZNF738, ZNRF1, ZNRF2, ZNRF3, ZRANB1, ZSWIM4, ZSWIM5, ZSWIM6, ZSWIM8, ZUP1, ZYG11A, ZYG11B |
| Prenylation | AIPL1, CHM, CHML, FNTA, FNTB, MUSK, PGGT1B, PLPP6, PTAR1, RABGGTA, RABGGTB, RCE1, CHM, COQ2, COX10, DHDDS, FDFT1, FDPS, FNTA, FNTB, GGPS1, NUS1, PDSS1, PDSS2, PGGT1B, PTAR1, RABGGTA, RABGGTB, UBIAD1 |
| Glycosylation | A4GALT, A4GNT, ABCA2, ACER2, ALG1, ALG10, ALG10B, ALG11, ALG12, ALG13, ALG14, ALG2, ALG3, ALG5, ALG6, ALG8, ALG9, B3GALNT1, B3GALNT2, B3GALT1, B3GALT2, B3GALT4, B3GALT5, B3GALT6, B3GALT9, B3GLCT, B3GNT2, B3GNT3, B3GNT4, B3GNT5, B3GNT6, B3GNT7, B3GNT8, B3GNT9, B4GALNT2, B4GALT1, B4GALT5, B4GALT7, B4GAT1, C1GALT1, C1GALT1C1, C1GALT1C1L, CHP1, CHST4, CRPPA, DAD1, DDOST, DERL3, DHDDS, DOLK, DOLPP1, DPAGT1, DPM1, DPM2, DPM3, DPY19L1, DPY19L2, DPY19L2P2, DPY19L3, DPY19L4, ENTPD5, EOGT, FKRP, FKTN, FUT3, FUT4, FUT5, FUT6, FUT7, FUT8, FUT9, GAL3ST1, GALNT1, GALNT10, GALNT11, GALNT12, GALNT13, GALNT14, GALNT15, GALNT16, GALNT17, GALNT18, GALNT2, GALNT3, GALNT4, GALNT5, GALNT6, GALNT7, GALNT8, GALNT9, GALNTL6, GCNT1, GCNT3, GCNT4, GFPT1, GFPT2, GOLGA2, GXYLT1, GXYLT2, IL15, KRTCAP2, LARGE1, LARGE2, MAGT1, MAN1A1, MAN1A2, MAN1C1, MAN2A2, MAN2B1, MAN2C1, MGAT1, MGAT2, MGAT3, MGAT4A, MGAT4B, MGAT4C, MGAT4D, MGAT5, MGAT5B, MLEC, MOGS, MPDU1, NUDT14, NUS1, OGA, OGT, OST4, OSTC, PGM3, PLOD1, PLOD2, PLOD3, PMM1, PMM2, POFUT1, POFUT2, POGLUT1, POGLUT2, POGLUT3, POMGNT1, POMGNT2, POMK, POMT1, POMT2, RAMP1, RFT1, RPN1, RPN2, RXYLT1, SLC35C1, SLC39A8, SLC51B, SRD5A3, ST3GAL1, ST3GAL2, ST3GAL3, ST3GAL4, ST6GAL1, ST6GALNAC2, ST6GALNAC4, ST8SIA4, ST8SIA6, STT3A, STT3B, TET1, TET2, TET3, TMEM165, TMEM258, TMTC1, TMTC2, TMTC3, TMTC4, TRAK1, TUSC3, UBE2J1, UGGT1, UGGT2, XXYLT1 |
| Myristoylation | ACAA1, HADHB, NMT1, NMT2, SCP2, ZDHHC15, ZDHHC17, ZDHHC2, ZDHHC20, ZDHHC3, ZDHHC7 |
| S-nitrosylation | ACE, ATP2B4, DMD, GAPDH, MIR132, MIR212, NCOA7, NOS1, NOS1AP, NOS2, OXR1, S100A8, S100A9, SNTA1, TBC1D24, TXN |
| Glutathionylation | ABCC1, ABCC10, ABCC11, ABCC2, ABCC3, ABCC4, ABCC6, AKR1A1, CHAC1, CHAC2, CNDP2, ESD, GCLC, GCLM, GGCT, GGT1, GGT5, GGT6, GGT7, GSS, GSTA1, GSTA2, GSTA3, GSTA4, GSTA5, GSTK1, GSTM1, GSTM2, GSTM3, GSTM4, GSTM5, GSTO1, GSTO2, GSTP1, GSTT1, GSTT2, GSTT2B, GSTZ1, HPGDS, MGST1, MGST2, MGST3, OPLAH, RALBP1 |
| SUMOylation | AAAS, AR, AURKA, AURKB, BIRC5, BLM, BMI1, BRCA1, CASP8AP2, CBX2, CBX4, CBX5, CBX8, CDCA8, CDKN2A, CETN2, CHD3, CREBBP, CTBP1, DAXX, DDX17, DDX5, DNMT1, DNMT3A, DNMT3B, EID3, EIF2AK2, EP300, ESR1, FOXL2, H4C1, H4C11, H4C12, H4C13, H4C14, H4C15, H4C16, H4C2, H4C3, H4C4, H4C5, H4C6, H4C8, H4C9, HDAC1, HDAC2, HDAC4, HDAC7, HERC2, HIC1, HIPK2, HNRNPC, HNRNPK, IKBKE, IKBKG, INCENP, ING2, L3MBTL2, MBD1, MDC1, MDM2, MITF, MRTFA, MTA1, NCOA1, NCOA2, NCOR2, NDC1, NFKB2, NFKBIA, NOP58, NPM1, NR1H2, NR1H3, NR1H4, NR1I2, NR2C1, NR3C1, NR3C2, NR4A2, NR5A1, NR5A2, NRIP1, NSMCE1, NSMCE2, NSMCE3, NSMCE4A, NUP107, NUP133, NUP153, NUP155, NUP160, NUP188, NUP205, NUP210, NUP214, NUP35, NUP37, NUP42, NUP43, NUP50, NUP54, NUP58, NUP62, NUP85, NUP88, NUP93, NUP98, PARK7, PARP1, PCGF2, PCNA, PGR, PHC1, PHC2, PHC3, PIAS1, PIAS2, PIAS3, PIAS4, PML, POM121, POM121C, PPARA, PPARG, PPARGC1A, RAD21, RAD52, RAE1, RANBP2, RANGAP1, RARA, RELA, RING1, RNF168, RNF2, RORA, RPA1, RWDD3, RXRA, SAE1, SAFB, SATB1, SATB2, SCMH1, SEC13, SEH1L, SENP1, SENP2, SENP5, SIN3A, SMC1A, SMC3, SMC5, SMC6, SP100, SP3, STAG1, STAG2, SUMO1, SUMO2, SUMO3, SUZ12, TDG, TFAP2A, TFAP2B, TFAP2C, THRA, THRB, TOP1, TOP2A, TOP2B, TOPORS, TP53, TP53BP1, TPR, TRIM27, TRIM28, UBA2, UBE2I, UHRF2, VDR, VHL, WRN, XPC, XRCC4, ZBED1, ZNF131, ZNF350 |
| Neddylation | AMER1, ANKRD9, ASB1, ASB10, ASB11, ASB12, ASB13, ASB14, ASB15, ASB16, ASB17, ASB18, ASB2, ASB3, ASB4, ASB5, ASB6, ASB7, ASB8, ASB9, BIRC5, BRCA1, BTBD1, BTBD6, BTRC, CAND1, CCDC22, CCDC8, CCNF, CDKN1A, CISH, COMMD1, COMMD10, COMMD2, COMMD3, COMMD4, COMMD5, COMMD6, COMMD7, COMMD8, COMMD9, COP1, COPS2, COPS3, COPS4, COPS5, COPS6, COPS7A, COPS7B, COPS8, CUL1, CUL2, CUL3, CUL4A, CUL4B, CUL5, CUL7, CUL9, DCAF10, DCAF11, DCAF13, DCAF16, DCAF17, DCAF4, DCAF5, DCAF6, DCAF7, DCAF8, DCUN1D1, DCUN1D2, DCUN1D3, DCUN1D4, DCUN1D5, DDA1, DDB1, DDB2, DPP3, DTL, ELOB, ELOC, EPAS1, ERCC8, FBXL12, FBXL13, FBXL14, FBXL15, FBXL16, FBXL18, FBXL19, FBXL20, FBXL22, FBXL3, FBXL4, FBXL5, FBXL7, FBXL8, FBXO10, FBXO11, FBXO15, FBXO17, FBXO2, FBXO21, FBXO22, FBXO27, FBXO30, FBXO31, FBXO32, FBXO4, FBXO40, FBXO41, FBXO44, FBXO6, FBXO7, FBXO9, FBXW10, FBXW11, FBXW12, FBXW2, FBXW4, FBXW5, FBXW7, FBXW8, FBXW9, FEM1A, FEM1B, FEM1C, GAN, GPS1, HIF1A, HIF3A, KBTBD13, KBTBD6, KBTBD7, KBTBD8, KCTD6, KCTD7, KEAP1, KLHL11, KLHL13, KLHL2, KLHL20, KLHL21, KLHL22, KLHL25, KLHL3, KLHL41, KLHL42, KLHL5, KLHL9, LMO7, LRR1, LRRC41, MUL1, NAE1, NEDD8, NEURL2, NFE2L2, NPLOC4, NUB1, OBSL1, PALB2, PSMA1, PSMA2, PSMA3, PSMA4, PSMA5, PSMA6, PSMA7, PSMA8, PSMB1, PSMB10, PSMB11, PSMB2, PSMB3, PSMB4, PSMB5, PSMB6, PSMB7, PSMB8, PSMB9, PSMC1, PSMC2, PSMC3, PSMC4, PSMC5, PSMC6, PSMD1, PSMD10, PSMD11, PSMD12, PSMD13, PSMD14, PSMD2, PSMD3, PSMD4, PSMD5, PSMD6, PSMD7, PSMD8, PSMD9, PSME1, PSME2, PSME3, PSME4, PSMF1, PUM2, RBBP5, RBBP7, RBX1, RNF7, RPS27A, SEM1, SENP8, SKP1, SKP2, SOCS2, SOCS3, SOCS5, SOCS6, SPSB1, SPSB2, SPSB3, SPSB4, SQSTM1, TULP4, UBA3, UBA52, UBB, UBC, UBD, UBE2D1, UBE2D2, UBE2D3, UBE2F, UBE2M, UBXN7, UCHL3, UFD1, VCP, VHL, WDR5, WDTC1, WSB1, WSB2, ZBTB16 |
| Succinylation | ACOT4, ALAS1, ALAS2, ALDH5A1, CPT1A, DLST, KAT2A, MMUT, NUDT19, NUDT7, NUDT8, OGDH, OGFH, SDHA, SDHAF3, SIRT5, SIRT7, SUCLA2, SUCLG1, SUCLG2 |
| Crotonylation | ACADS, ACOX1, ACOX2, ACOX3, ACSS2, AF9, BRD4, CBP, CDYL, DPF2, ECHS1, EP300, EPB41L4A-AS, ESA1, FOSIR5, GCDH, GCN5, GNAT, HAT1, HBO1, HDAC1, HDAC2, HDAC3, HDAC6, HDAC7, HDAC8, KAT7, MEAF6, MLLT3, MLLT4, MOF, MOZ, NEZAT1, PCAF, RTT109, SIRT1, SIRT2, SIRT3, SIRT6, SIRT7, TAF1, TAF14, TIP60, YEATS2, YEATS4 |
| Beta-hydroxybutyryration | EP300, HDAC1, HDAC2 |
| Lactylation | CREBBP, EMB, EP300, GLO1, HAGH, HDAC1, HDAC2, HDAC3, HDAC8, LDHA, LDHAL6A, LDHAL6B, LDHB, LDHC, LDHD, SIRT1, SIRT2, SIRT3, SLC16A1, SLC16A3, SLC16A7, SLC16A8, SLC5A12, SLC5A8 |

Supplement Table2 The scores of PTMs-related prognostic genes*.*

| Gene | Score |
| --- | --- |
| *TUBA1C* | 9.401968 |
| *FBXO39* | 6.887073 |
| *ABCC3* | 5.868274 |
| *FBXO17* | 8.508007 |
| *GALNT5* | 4.853204 |
| *DPY19L1* | 6.022164 |
| *SOCS1* | 6.910487 |
| *CPA4* | 6.224788 |
| *NNMT* | 5.497615 |
| *TOM1L1* | 7.762546 |
| *ZDHHC22* | -6.57669 |
